# Dosage compensation in the process of inactivation/reactivation during both germ cell development and early embryogenesis in mouse

**DOI:** 10.1038/s41598-017-03829-z

**Published:** 2017-06-16

**Authors:** Xiaoyong Li, Zhiqiang Hu, Xuelin Yu, Chen Zhang, Binbin Ma, Lin He, Chaochun Wei, Ji Wu

**Affiliations:** 1 Renji Hospital, Key Laboratory for the Genetics of Developmental & Neuropsychiatric Disorders (Ministry of Education), Bio-X Institutes, School of Medicine, Shanghai Jiao Tong University, Shanghai, 200240 China; 2 School of Life Sciences and Biotechnology, Shanghai Jiao Tong University, 800 Dongchuan Road, 200240 Shanghai, China; 3 Shanghai Center for Bioinformation Technology, 1278 Keyuan Road, Pudong District, Shanghai 201203 China; 4 Key Laboratory of Fertility Preservation and Maintenance of Ministry of Education, Ningxia Medical University, Yinchuan, 750004 China

## Abstract

Ohno proposed that dosage compensation in mammals evolved as a two-step mechanism involving X-inactivation and X-upregulation. While X-inactivation is well characterized, it remains to further analysis whether upregulation of the single activated X chromosome in mammals occurs. We obtained RNA-seq data, including single-cell RNA-seq data, from cells undergoing inactivation/reactivation in both germ cell development and early embryogenesis stages in mouse and calculated the X: A ratio from the gene expression. Our results showed that the X: A ratio is always 1, regardless of the number of X chromosomes being transcribed for expressed genes. Furthermore, the single-cell RNA-seq data across individual cells of mouse preimplantation embryos of mixed backgrounds indicated that strain-specific SNPs could be used to distinguish transcription from maternal and paternal chromosomes and further showed that when the paternal was inactivated, the average gene dosage of the active maternal X chromosome was increased to restore the balance between the X chromosome and autosomes. In conclusion, our analysis of RNA-seq data (particularly single-cell RNA-seq) from cells undergoing the process of inactivation/reactivation provides direct evidence that the average gene dosage of the single active X chromosome is upregulated to achieve a similar level to that of two active X chromosomes and autosomes present in two copies.

## Introduction

The formation of sex was one of the most important events in evolution. Sex in mammals is determined by the sex chromosomes. While males and females have two copies of each autosomes, the sex chromosomes are different. In therian mammals, females have two matching X chromosomes (XX), while males have two distinct sex chromosomes (X and Y). In females, one of the two X chromosomes is inactivated in females, whereas in males, the Y-chromosome has lost most of its genes. Thus, both sexes have one active allele per sex chromosome gene but two active alleles per autosomal gene^1, 2^. In 1967, Susumu Ohno proposed the dosage compensation theory to explain the gene dosage imbalance between sex chromosomes and autosomes (Ohno’s hypothesis)^3^. The dosage compensation theory can be divided into two processes. The first process involves the silencing of one female X chromosome (X chromosome inactivation, XCI or Xi) to balance the X-dosage with that of the male (X-inactivation)^4, 5^. This process has been extensively studied at the mechanistic and evolutionary levels. The second process involves a two-fold hyper activation of the X chromosome in both sexes (X-upregulation)^3^. However, Ohno’s hypothesis remains controversial, and this study presents an additional analysis using both new and existing data.

Early microarray studies have provided evidence supporting Ohno’s hypothesis. These studies showed that the overall expression level of genes in the X chromosome was twice that of genes in autosomes (X: AA ≈1)^6–10^. However, this result was first challenged by mRNA-seq study^11^. This study used mRNA-seq data to calculate the X: AA ratios and obtained values that were approximately 0.5 in a variety of human tissues, indicating that dosage compensation did not occur in the active X chromosome, thereby rejecting Ohno’s hypothesis. Several subsequent studies challenged this analysis and supported Ohno’s hypothesis^12–14^. Furthermore, many studies have attempted to explain this ongoing controversy, with some studies supporting Ohno’s hypothesis and other studies contradicting it^15–19^. One way to evaluate the existence of X-upregulation is to compare the expression of X-linked genes in mammals to that of “ancestral” genes in chickens. This study concludes that there is no evidence of X-upregulation in placental mammals. However, similar techniques showed an X-upregulation in the male opossum and oldest X chromosome in marsupials^15, 16^. In addition, this comparative study may address evolutionary aspects of X expression it remains unclear whether contemporary mammals can be directly compared to contemporary birds with different physiology^17^. Following an analysis of human proteomic data from 22 tissues, it was reported that X-upregulation is absent at the protein level, indicating that Ohno’s hypothesis is also invalid at the protein level^18^. An analysis of the genes that encode components of large protein complexes (≥7 members) revealed that their expressions, which were expected to be dosage-sensitive, were similar to those of autosomal genes within the complex. These results support Ohno’s hypothesis that X chromosome inactivation acts as a dosage-compensation mechanism^19^. Importantly, Marks *et al*. found that gene expression in the active X chromosome (Xa) is upregulated, resulting in complete dosage compensation between X-linked and autosomal genes in the early stages of XCI during the differentiation of female embryonic stem cells (ESCs) to embryoid bodies (EBs)^20^. Nevertheless, Ohno’s hypothesis is still debated^21, 22^.

However, the status of the process of inactivation/reactivation during germ cell development and early embryogenesis in mouse is not well characterized. There are many types of cells involved with inactivation/reactivation, including the cells with two active X chromosomes, cells with one active X chromosome and the cells with one active X chromosome and one X chromosome partly inactivated or reactivated. First, the inactivated X chromosome is reactivated between embryonic day (E) 8.5 and E12.5 during the primordial germ cell (PGC) development in female mouse^23–25^. Furthermore, X-reactivation proceeds gradually in a stepwise manner. After E10.5, the reactivation of X-linked genes significantly accelerates, and most of the X-linked genes tested showed biallelic expression at E12.5^26^. In contrast, our previous studies have shown that female germline stem cells (FGSCs) exist in postnatal mouse ovaries^27–30^, indicating that not all oogonia enter meiosis. Second, two types of X-chromosome inactivation (XCI) occur during female embryonic development in mouse. One type involves random XCI, which is observed in cells derived from epiblasts, when one of the two X chromosomes (paternal or maternal) is randomly inactivated. The other type involves imprinted X chromosome inactivation (iXCI), which is observed in extra-embryonic tissues and causes XCI of the paternal X chromosome (Xp)^31^. The initiation of iXCI begins in early preimplantation embryos and the Xp gene *Xist* is expressed around the four-cell stage^32^. Moreover, the inactive paternal X chromosome is reactivated in the blastocyst. One of the two chromosomes will randomly loses activity later, which is never regained in the offspring^33, 34^.

In the present study, we focused on the above process of inactivation/reactivation in both germ cell development and early embryogenesis in mouse. We used RNA-seq data from mouse germ cells at different developmental stages and female embryonic stem cells to compare the expression levels of X-linked and autosomal genes. We concluded that it is not suitable to include genes with no/low expression when measuring the X: A ratio (The ratio of X-linked to autosomal expression as measured by RNA-seq, namely, the X: A ratio is indicative of the X: AA ratio when the cell contains one active X chromosome and indicative of the XX: AA ratio when the cell contains two active X chromosomes). We also found that the average gene expression in the X chromosome always achieved a similar level to that in autosomes and that the median X: A expression ratio is approximately 1 whether there are one or two active X chromosomes present in cells. Additionally, when single-cell RNA-seq data were analyzed by excluding no-/low-expression genes, our results also supported Ohno’s hypothesis. Ultimately, the analysis of single-cell RNA-seq data from individual cells of mouse preimplantation embryos of mixed backgrounds that we could use strain-specific SNPs to distinguish transcription from the maternal and paternal chromosomes provided direct evidence for dosage compensation. When the paternal X chromosome was inactivated, the average gene dosage in the active maternal X chromosome increased to achieve a balance between the X chromosome and autosomes.

## Results

### Dosage compensation in the process of reactivation during mouse germ cell development

**Figure 1:**
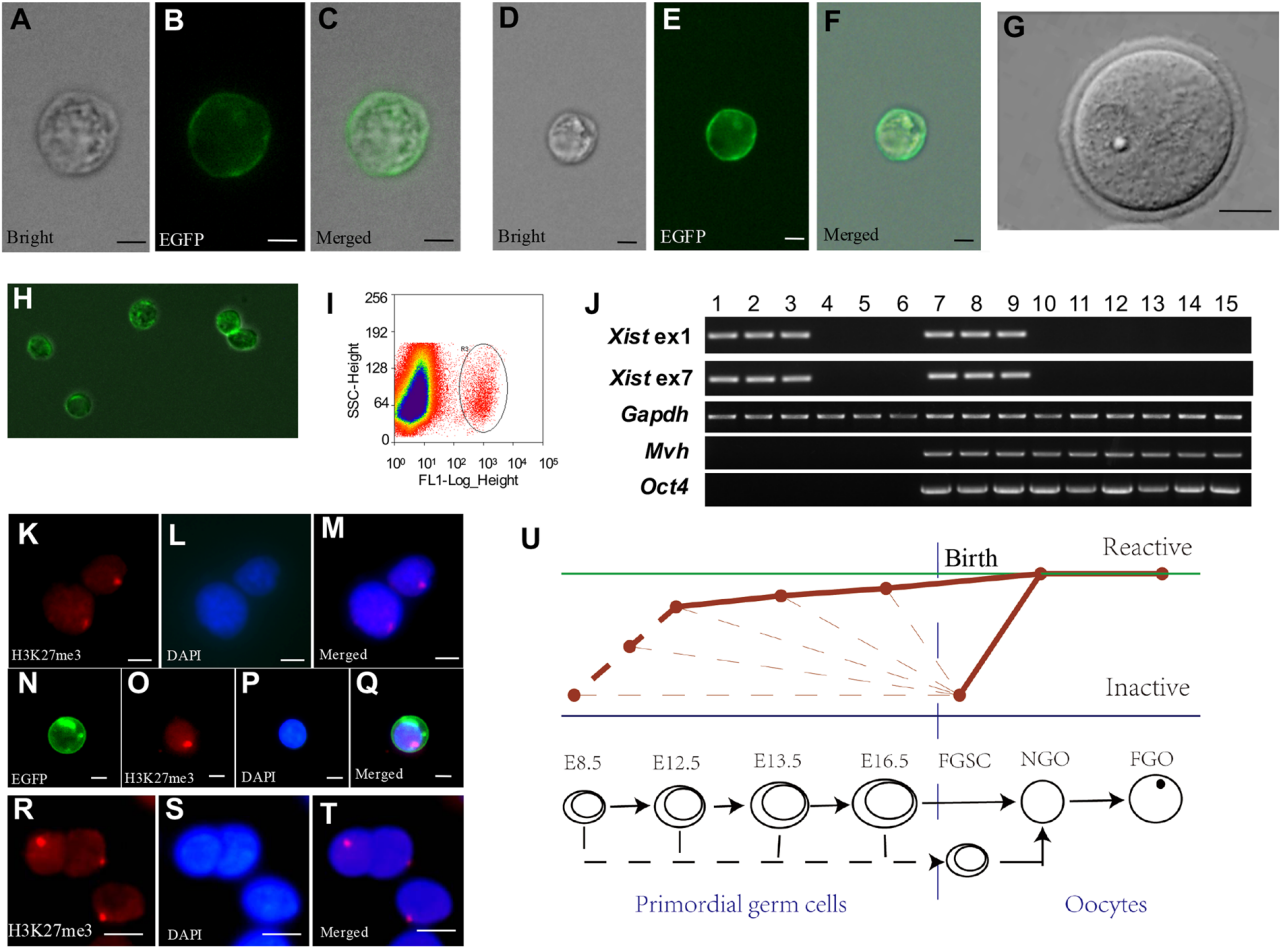
The X chromosome undergoes a process of reactivation in female germ cells from FGSCs to oocytes. (**A**–**C**) Representative morphology of Mvh positive cells from XX genital ridges after E12.5 under light microscopy and fluorescence microscopy. (**D**–**F**) Representative images of Mvh positive cells from ovaries of 3–5 days old Mvh-GFP transgenic mice (Mvh-cre; ROSA^mT/mG^ mice) under light microscopy and fluorescence microscopy. (**G**) Representative morphology of oocytes. (**H**) Representative view of merger for Mvh positive cells under light microscopy and fluorescence microscopy after purification with FACS.(**I**) An example for FGSC purification with FACS. (**J**) Single cell RT-PCR analysis of *Xist*, *Mvh* and *Oct4*. Lane 1–3, female Tail fibroblasts (positive control); lane 4–6, male tail fibroblasts (negative control); lane 7–9, FGSCs; lane 10–12, NGO; lane 13–15, FGO. Full-length gels are presented in Supplementary Fig. S15. (**K**–**M**) FGSC cell line showed the expected frequencies of the Xi-like enrichment for H3K27me3. Cells were counterstained with DAPI. (**N**–**Q**) Fresh isolated FGSCs showed the expected frequencies of the Xi-like enrichment for H3K27me3. Cells were counterstained with DAPI. (**R**–**T**) Female tail fibroblasts showed the expected frequencies of the Xi-like enrichment for H3K27me3 (positive control). Cells were counterstained with DAPI. (**U**)Schematic illustration of the process of reactivation in germ cell development: the FGSCs, PGCs and oocytes formed a process from X inactivation to X reactivation in germ cell development. The solid line shows the process we determined, while the dotted line shows the process we do not test. The slim dotted line indicated that early germ cells contain the cells entering meiosis and very small amount of the cells not entering meiosis (or precursors of FGSCs). For (**A**–**F**,**H**,**K**–**T**), Scale bar = 5 μm, for G, Scale bar = 20 μm. The experiments were conducted three times.

To examine whether the X chromosome undergoes a process of reactivation in mouse female germ cells at the different stages examined in this study, we isolated PGCs (E12.5, E13.5 and E16.5) and FGSCs using two-step enzymatic digestion, as previously described^27^, and then purified them using fluorescence-activated cell sorting (FACS) (Fig. 1A–F,H,I). We also collected oocytes (Fig. 1G). Next, the germ cells collected at different stages were analyzed using single-cell reverse-transcriptase PCR (RT-PCR) for *Xist* (an initial factor of X chromosome inactivation and the hallmark of an inactive X chromosome^35–38^), *Oct4* (a germ cell-specific transcription factor^39^) and *Mvh* (which is exclusively expressed in germ cells^40^). Tail fibroblasts isolated from mice were used as controls. The results showed that in all FGSCs and in 2 of 83 E12.5 PGCs, 2 of 109 E13.5 PGCs and 1 of 87 E16.5 PGCs, *Xist* was expressed. In contrast, oocytes and most of the PGCs did not express *Xist* (Figs 1J and S1–S3). These results demonstrated that one of the X-chromosomes in FGSCs was inactive. To confirm this finding, the frequencies of the Xi-like enrichment for histone H3 trimethylated at lysine 27 (H3k27me3)(a mark of transcriptionally silent chromatin^41^) in the FGSC line^27^ and FGSCs isolated from MVH-GFP mice was determined using immunofluorescence analysis. Our result showed the expected frequencies of the Xi-like enrichment for H3K37me3 in FGSCs (Fig. 1K–T). Taken together, these results indicated that the X-chromosome undergoes a process of reactivation in female germ cells from FGSCs to oocytes (Fig. 1U). More precisely, one of the two X chromosomes in FGSCs is inactivated and the other X chromosome is reactivated in most of the E12.5 PGCs, E13.5 PGCs, and E16.5 PGCs. Moreover, oocytes contain two active X-chromosomes^42, 43^.

**Figure 2:**
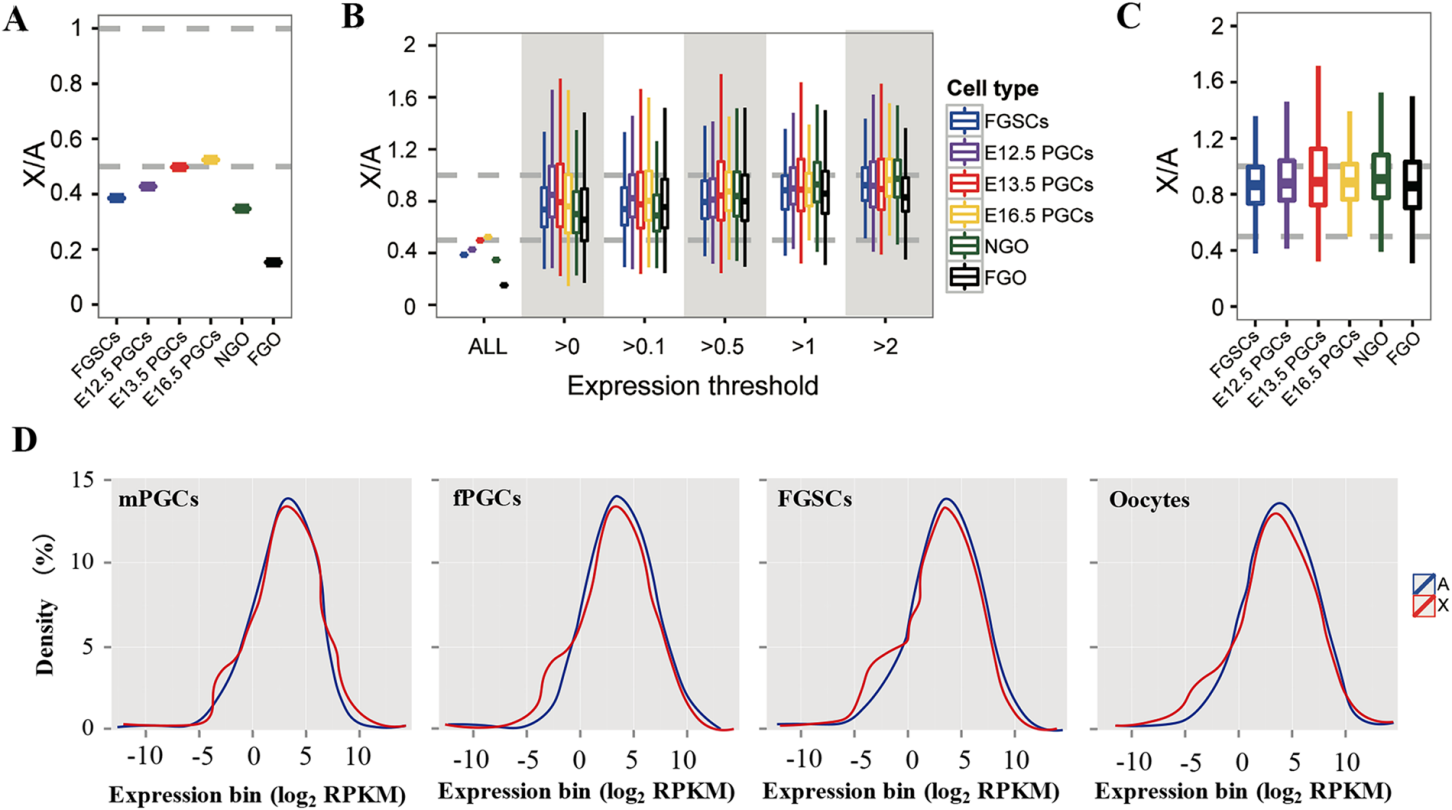
Comparisons of RNA-Seq gene expression levels between the X chromosome and autosomes in germ cell development. (**A**) X: A ratios of median expressions from germ cells when genes with no or very low expression are taken into consideration. The X: A ratio is significant closer to 0.5 compared to1 (Student’s t-test, p < 0.001). (**B**) For all types of germ cells, the X: A median expression ratios increased to close to 1 as no-/low-expression genes were gradually removed and remained stable after genes with FPKM ≤ 1 were removed. There were no differences between each types of germ cells. (P > 0.05, by Student’s t-test). (**C**) X: AA ratios of median expressions from germ cells when excluding no-/low-expression genes. There were no differences between each types of germ cells. (P > 0.05, by Student’s t-test) and the X: A ratio is significant closer to 1 compared to 0.5 (student’s t-test, p < 0.001). (**D**) Histograms of X-linked and autosomal expression distributions for male and female PGCs (mPGCs and fPGCs), FGSCs and NGO (Oocytes) (P > 0.05, by Kolmogorov-Smirnov test). The percentiles in all boxplots are 0.05, 0.25, 0.5, 0.75 and 0.95.

On the basis of the above information, we used RNA-seq data from FGSCs, PGCs, non-growing oocytes (NGOs) and fully grown oocytes (FGOs) to compare the expression levels of X-linked genes and autosomal genes in germ cell development in mouse. The RNA-Seq data for E12.5 PGCs, FGSCs and FGO produced in our laboratory provided sufficient depth of reads (Supplementary Table S1). Additionally, there were no statistically significant differences between the RNA-seq data produced in our laboratory and the data obtained from public database (Student’s t-test, p > 0.05. Supplementary Fig. S4). Therefore, we used these data to calculate dosage compensation states in germ cell development. Our results showed that the ratios of X-linked expression to autosomal expression, as measured by RNA-seq, were close to or less than 0.5 when all genes were taken into consideration for all types of germ cells (Fig. 2A, Table 1). The median X: A expression ratio was close to or less than 0.5 in cells with one active and one inactive X chromosome (FGSCs) as well as in cells with two active X chromosomes (E12.5 PGCs, E13.5 PGCs, E16.5 PGCs, NGO, and FGO) (Fig. 2A, Table 1). This finding was puzzling in that such a low X: A (XX: AA ratio) was observed in these cells despite them having twice the transcriptional output from the X-chromosomes. In addition, male and female E13.5 PGCs showed the similar results, the X: A ratios for both PGCs were approximately 0.5 (Supplementary Fig. S5). A previous study showed that the X: A ratios were approximately 0.5 when all genes were taken into consideration in human and mouse tissues and concluded that no dosage compensation occurred in the active X chromosome^11^. However, our analysis of specific germ cells and female mouse embryonic stem cells containing two active X chromosomes also demonstrated a similar X: A ratio. Thus, it appears to be inappropriate to calculate the X: A ratio when all genes are taken into consideration.

**Table 1:** The median X: A expression ratio in female germ cells at the different stages.

Cell type	Number of active X chromosome	X: A ratio (all genes)	X: A ratio (expression genes)
FGSCs	1	0.39^£^	0.89^§^
E12.5 PGCs	≈2	0.48^£^	0.91^§^
E13.5 PGCs	≈2	0.49^£^	0.93^§^
E16.5 PGCs	≈2	0.52^£^	0.93^§^
NGO	2	0.38^£^	0.94^§^
FGO	2	0.15^£^	0.85^§^

^£^Means the X: A ratio is significant close to 0.5 compare to1 (χ-squared test, p < 0.001), ^§^means the X: A ratio is significant close to 1 compare to 0.5 (χ-squared test, p < 0.001).

We can imagine that when the dosage of genes with no expression (0 fragments per kilobase of exon per million mapped fragments (FPKM)) is doubled, the dosage remains unchanged (still 0 FPKM). However, when the active genes are different, the dosage will be twice as high. From this perspective, genes with no expression (0 FPKM) should be excluded when evaluating dosage compensation. On the other hand, the low-abundance transcripts might result from technical or biological noise rather than active transcripts. Moreover, most RNA-seq experiments do not provide sufficient read depth to generate high-confidence estimates of gene expression for low-abundance transcripts. Consequently, the community adopted several heuristics for RNA-seq analysis, most notably an arbitrary expression threshold of 0.3–1 FPKM for downstream analysis^44–46^, which may explain the puzzling X: A ratios observed when the expression levels of X-linked genes and autosomal genes during germ cell development were compared. To confirm that low-abundance transcripts might result from technical or biological noise rather than active transcripts, forty genes with low-expression (FPKM < 1) in FGSCs but high-expression (FPKM > 1) in PGCs were randomly selected from our RNA-seq data. We used RT-PCR to examine whether these low-expression genes resulted from technical or biological noise rather than active transcripts. Our results showed that 87.5% (35/40) of selected genes were not active transcripts (Supplementary Fig. S6).

Thus, we reanalyzed the above RNA-seq data with the no-/low-expression genes excluded. Before we filtered out the genes with low expression level, we would like to know the percentages of expressed genes that were left after each level of filtering. Our results showed that most of the expressed genes were left after filtering (Supplementary Table S2). The calculated ratios of X-linked expression to autosomal expression based on RNA-seq data were strongly dependent on the inclusion or removal of genes with no/low expression genes. For all types of germ cells, the X: A median expression ratios increased to approximately 1 as more no-/low-expression genes were removed and remained stable after the removal of genes with an FPKM < 1 (Fig. 2B, Table 1). Meanwhile the X: A median expression ratios were not statistically significant differences between each types of germ cells (P > 0.05, by Student’s t-test, Fig. 2C, Table 1). Moreover, the median X: A expression ratio for each type of cell in the germ cell development was independent of the number of active X chromosomes present in cell. Likewise, mouse male and female E13.5 PGCs yielded the same results; the median X: A expression ratios increased to approximately 1 as more no-/low-expression genes were removed and remained stable after the removal of genes with an FPKM < 1 for both male and female E13.5 PGCs (Supplementary Fig. S5). In addition, the absolute quantification of gene expression in the X chromosomes remained similar across all germ cells (Supplementary Fig. S7). Furthermore, the distributions of gene expressions were also similar between the X-chromosome and autosomes in male and female PGCS, FGSCs and Oocytes (P > 0.05, by Student’s t-test, Fig. 2D). The absence of doubling transcriptional output from the X-chromosome in these germ cells maintained a balanced expression with the autosomes. Moreover, when one of the X chromosomes was inactivated (female) or when only a single X chromosome (male) was present in the cell, the average gene dosage on the active X chromosome was upregulated to maintain a balanced expression with the autosomes. Additionally, the X: A ratios calculated using a previously published RNA-seq dataset from the early stages of XCI during the differentiation of female (ESCs) to embryoid bodies^20^ was in accordance with our finding by and large (Supplementary Fig. S8).

In conclusion, we demonstrated that no-/low-expression genes should be excluded when calculating the X: A ratio. Furthermore, our analysis demonstrated that there is a similar X: A ratio whether there are one or two active X chromosomes present in cells (the X: A ratio in all cell types is approximately 0.5 if no-/low-expression genes are included in the analysis, and approximately 1 if no-/low-expression are excluded). The combined analyses of mouse germ cells and embryonic stem cells supported the hypothesis that most of the expressed genes on the single active X chromosome were upregulated to achieve a similar level of expression to that of two active X chromosomes and autosomes present in two copies.

### Recent RNA-seq data obtained from mammalian tissues confirmed dosage compensation

Only a small number of species have been investigated for their dosage compensation status. Now, we analyzed the recent RNA-seq data obtained from mammalian tissues (seven *Rattus norvegicus* tissues, seven *Sus scrofa* tissues, eight *Bos taurus* tissues and eight *Macaca mulatta* tissues) and excluded no-/low-expression genes. For all cases, the X: A ratios were close to 1 for genes with an FPKM ≥ 1 (Student’s t-test, p < 0.05. Supplementary Fig. S8). These results indicated that the transcriptional output from the X chromosome was doubled in all species examined to achieve dosage compensation. The analyzed tissues were derived from both males and females, and similar X: A ratios were calculated for male and female tissues from all species examined. These results indicate that status of dosage compensation in *Rattus norvegicus*, *Sus scrofa*, *Bos Taurus* and *Macaca mulatta* is similar to that in *Homo sapiens* and *Mus musculus*.

### Single-cell RNA-Seq data confirms X-upregulation in mouse

Although the transcriptome data from various tissues supported Ohno’s hypothesis, further evidence was obtained from the RNA-seq analysis of tissues comprising many cell types. The single cell is the basic functional unit of life and dosage compensation is a biological phenomenon that occurs at the level of at single cell. Increasing experimental evidence and relevant theoretical derivations have shown that differences between individual cells can lead to important and even decisive consequences in many biological processes, including the occurrence and development of diseases. As a powerful tool to analyze global gene expression at the single-cell level, single-cell transcriptome sequencing (single-cell RNA-seq) enables the measurement of expressions of a single copy of the genome and can remove the effects of mixed expressions of multiple genomes in traditional tissue sequencing^47^. This tool can provide new insights into cell differentiation, cell-to-cell variation and gene regulation, and the inter dependencies between them.

**Figure 3:**
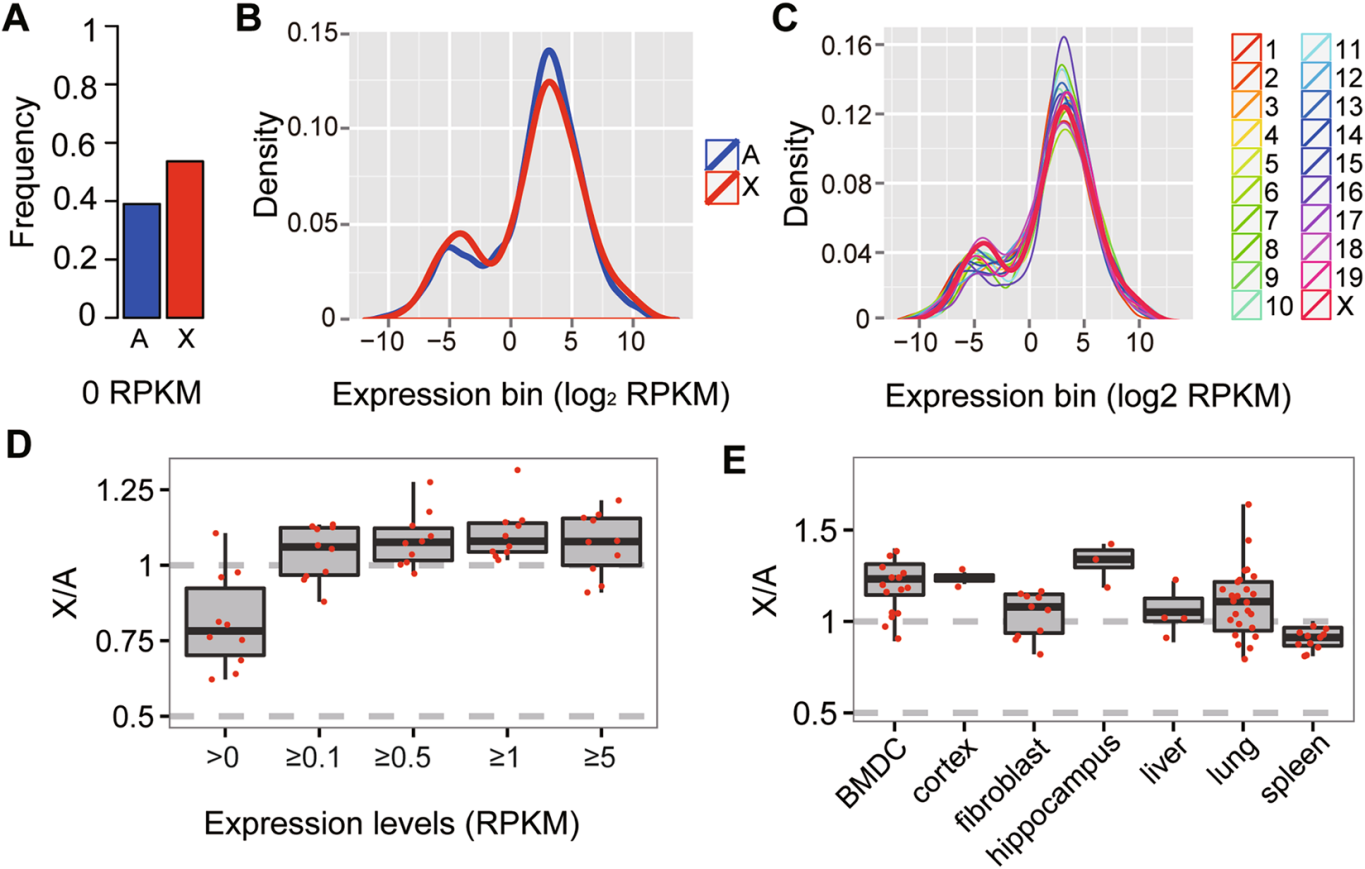
Distributions of gene expression are similar between the X chromosome and autosome in mammals at the single cell level. (**A**) The frequency of genes with no expression was significantly higher on the X chromosome than on autosomes in mouse fibroblast cell based on single cell RNA-Seq(P < 0.05, by Student’s t-test). (**B**) Histograms of X-linked and autosomal expression distributions (P > 0.05, by Kolmogorov-Smirnov test). (**C**) Histograms of expression distribution for genes with >0 FPKM on each mouse fibroblast chromosome expect (P > 0.05, by Kolmogorov-Smirnov test). (**D**) X: A median expression ratios based on single cell RNA-Seq data were strongly dependent on FPKM cutoffs (>0, ≥0.1, ≥0.5, ≥1, ≥5). There were no differences between each ≥0.1expression threshold. (P > 0.05, by Student’s t-test). (**E**) X: A ratios of median expressions based on single cell RNA-Seq from bone marrow-derived dendritic cells (BMDC) (male, N = 15), cortex cells (N = 2), fibroblast cells (female, N = 10), hippocampus cells (N = 3), liver cells (female, N = 4), lung cells (N = 25) and spleen cells (male, N = 10) when excluding no-/low-expression genes. Sex information is listed in parentheses after the cell names, and N represents the number of single cell RNA-seq data with this type of cells in the parentheses after the cell names. There were no differences between each types of single cells. (P > 0.05, by student’s t-test) except cortex and hippocampus (P < 0.05, by Student’s t-test). And the X: A ratio is significant closer to 1 compared to 0.5 (Student’s t-test, p < 0.001). The percentiles in all boxplots are 0.05, 0.25, 0.5, 0.75 and 0.95.

To determine whether X-linked genes were expressed at levels twice that of autosomal genes per active allele, we first investigated whether the X chromosome and autosomes had similar expression profiles at the single-cell level. We used single-cell RNA-seq data from mouse fibroblasts as an example to determine the distribution of X-linked and autosomal gene expression. In all mouse single fibroblast cells examined, the frequency of genes with no expression was significantly higher in the X-chromosome than in autosomes (P < 0.05, by Student’s t-test, Fig. 3A). The distributions of gene expressions were also similar between the X chromosome and autosomes in a single fibroblast cell, even when including all genes with any evidence of expression (>0 FPKM, Fig. 3B,C, Supplementary Fig. S10). Likewise, the calculated median X: A expression ratios based on single-cell RNA-seq data were strongly dependent on the inclusion or removal of genes with no/low expression, and remained stable after the removal of genes that were expressed weakly or not at all (Fig. 3D, Supplementary Table S3). Our results indicated that we could use FPKM < 0.1 as cutoff criteria in single cell RNA-seq data (Fig. 3D, Supplementary Table S3). We also showed that most of the expressed genes were not removed by the filter (Supplementary Table S4).

We further calculated the X: A ratios by excluding no-/low-expression genes (FPKM < 0.1) with several single-cell sequencing datasets. We found that the X: A ratios were all approximately1 (χ-squared test, Fig. 3E, Supplementary Table S3), which further supported the hypothesis that X-linked genes were upregulated to balance the expression of autosomes. Similar to previous studies in mammalian somatic tissues^6, 11, 12^, we found that the X: A ratios were higher in cells derived from brain tissue (cortex and hippocampus) compared to other tissues. We obtained similar results when we used a FPKM cutoff of 1 to compared the X: A ratio across different samples from single cell RNA-Seq data (Supplementary Fig. S10).The higher X: A ratio in the brain versus other tissues is independent of mammalian species and sex; however, the explanation for the higher X: A ratio should be confirmed by additional analyses. Nevertheless, we concluded that the median expression of genes from the single active X chromosome is doubled in the tissues to achieve dosage compensation at the single-cell level.

### Dosage compensation in the process of inactivation during mouse early embryogenesis using single-cell RNA-seq data

**Figure 4:**
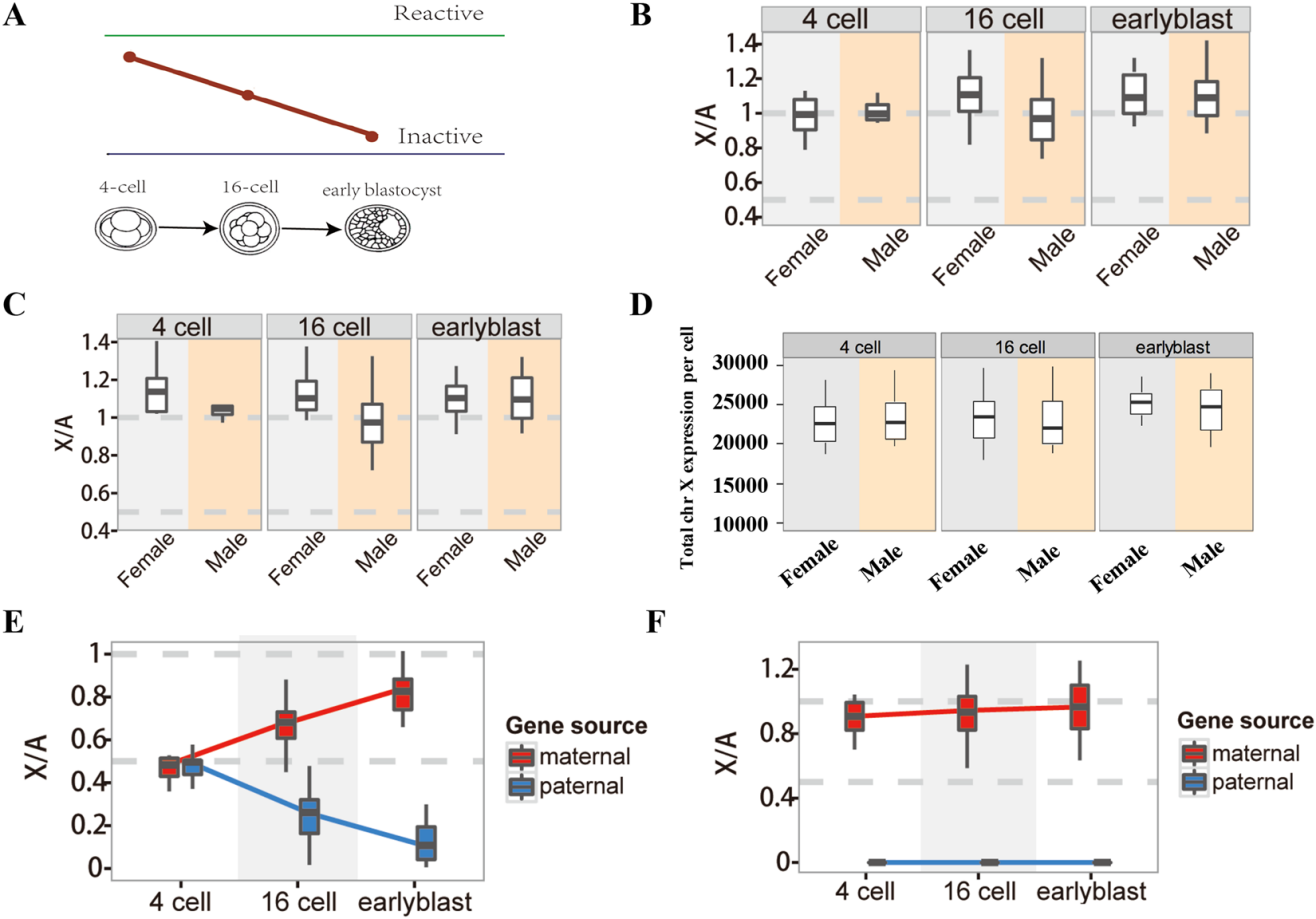
Comparisons of RNA-Seq gene expression levels between the X chromosome and autosomes in mouse early embryogenesis. (**A**) Schematic illustration of the process of inactivation in mouse early embryogenesis: The initiation of iXCI begins at early preimplantation in embryos and Xp-Xist is expressed around the four-cell stage. Next, the active paternal X chromosome becomes gradually inactive from the four-cell stage to early blastocyte stage to form a process from X reactivation to X inactivation. (**B**) X: A ratios of median expressions according to all reads for each sex and embryonic day. There were no differences between each types of embryonic cells and between males and females. (P > 0.05, by Student’s t-test). (**C**) X: AA ratios of median expressions according to SNP-containing reads for each sex and embryonic day. There were no differences between each types of embryonic cells and between males and females. (P > 0.05, by Student’s t-test). (**D**) Boxplots showing the distribution of cellular X chromosome FPKM sums for each sex and embryonic day. There were no differences between each types of embryonic cells and between males and females. (P > 0.05, by Student’s t-test). (**E**) Comparisons of X: AA ratios of median expressions between paternal X chromosome and maternal X chromosome from female 4-cell stage (N = 11), 16-cell stage (N = 21) and early blastocyst stage (N = 15) in female embryo. (**F**) Comparisons of X: AA ratios of median expressions between paternal X chromosome and maternal X chromosome from male 4-cell stage (N = 3), 16-cell stage (N = 27) and early blastocyst stage (N = 28) in male embryo. N represents the number of single cell RNA-seq data with this stage in the parentheses after early embryogenesis. The percentiles in all boxplots are 0.05, 0.25, 0.5, 0.75 and 0.95.

Based on information that the X-chromosome undergoes a process of inactivation in mouse early embryogenesis: at the four-cell stage both X chromosomes are active, but the paternal X is inactivated in preimplantation development^32^ (Fig. 4A). We examined a time-series of transcriptome sequencing data to obtain more insight into this developmental process. We used recently published single-cell RNA-seq data in which all cells were isolated *in vivo* from F1 embryos (CAST/EiJ × C57BL/6J, abbreviated as CAST and C57, respectively), from the oocyte to blastocyst stages of mouse preimplantation development. CAST and C57 mice are relatively divergent, and thus, this F1 hybrid is rich in SNPs (82% of all genes expressed during preimplantation development contained ≥1 informative SNP and different SNPs within the same gene gave coherent allelic calls), permitting the use of strain-specific SNPs to distinguish transcription from maternal (Xm) and paternal chromosomes (Xp)^33^. To assess allele-specific expression using SNPs with this RNA-seq data, we first showed that the SNP-containing genes were widely scattered on all chromosomes and that the ratio (SNP-containing genes/total genes) from each chromosome was very similar (Supplementary Fig. S11A). Meanwhile, we also found that SNP-containing genes were widely scattered on each level of filtering and that the ratio (SNPs containing expression genes/total expression genes in this filtering level) from each level of filtering was very similar (Supplementary Fig. S11B). What’s more, although not all genes contained strain-specific SNPs, we found that (i) the X: A ratios calculated according to all reads were similar to SNP-containing reads, (ii) the X: A ratio in mouse early embryogenesis from the four-cell stage to the early blastocyst stage in both sexes were close to 1 (similar to the results from germ cell development), (iii) the X: A ratio is the same in cell types in which X chromosomes inactivation has not yet taken place and also in cell types with inactive X chromosomes (Fig. 4B,C), indicating that we could use SNP-containing reads to describe the expression landscapes. The median X: A ratios were no statistically significant differences between each types of embryonic cells and between males and females (P > 0.05, by Student’s t-test). To confirm this, we compared gene expression of the X chromosome between each embryonic cell type. Our results showed that the absolute quantification of X chromosome gene expression remained similar in each embryonic cell type in mouse (Supplementary Fig. S12). In addition, we found that the total gene expression of the X chromosome per cell were no statistically significant differences between each embryonic cell type or between males and females (P > 0.05, by student’s t-test, Fig. 4D).

To investigate the transcriptional changes that occur on the Xi and Xa specifically, we calculated the ratio of X_p_: AA and X_m_: AA over the time course. In the four-cell stage, the X_p_: AA ratios and X_m_: AA ratios of female embryos were both approximately 0.5 (P > 0.05, by student’s t-test, Fig. 4E, Supplementary Table S5), indicating that both the X_p_ and X_m_ chromosomes were active, consistent with previous research^32^. In the 16-cell stage, the X_m_: AA and X_p_: AA ratios of female embryos were 0.69 and 0.30, respectively (Fig. 4E, Supplementary Table S5). This result indicated that the average gene dosage on the maternal X chromosome was increased to compensate for the decreased gene dosage on the paternal X chromosome, which ensured that the X: A ratio remained close to 1. In the early blastocyst stage, the X_m_: AA and X_p_: AA ratios of female embryos were 0.84 and 0.12, respectively (Fig. 4E, Supplementary Table S5), which revealed a continuous process of inactivation of the paternal X chromosome and dosage compensation in the maternal X chromosome to ensure that the X: A ratio remained close to 1. Furthermore, male embryos only have one maternal X chromosome and their X_m_: AA ratios were close to 1 (the X_p_: AA ratio was 0) for all stages from the four-cell stage to the early blastocyst stage (Fig. 4F, Supplementary Table S5), indicating that the the average gene dosage on the single active X chromosome was upregulated to achieve a similar level to that of the autosomes, which are present in two copies.

Our comparison of X: A ratios in mouse early embryogenesis represented direct evidence of dosage compensation and provided a visual representation that the average gene expression level from the active X chromosome increased during the process of inactivation of the other X chromosome. In addition, the increase of the average gene dosage on the active X chromosome was dependent on the decrease of the average gene dosage on the inactive X chromosome, such that the X: A ratio remained close to 1. Taken together, these results represent direct evidence to support the hypothesis that X-linked genes are upregulated to match the expression level of autosomes.

## Discussion

Dosage compensation is the fundamental process by which gene expression from the male monosomic X chromosome and from the diploid set of autosomes is equalized. Dosage compensation exists widely in the biosphere and is a complex phenomenon that includes different mechanisms^38, 39, 48, 49^. During the evolution of the X chromosomes from a pair of autosomes, the gene expressions of the X chromosomes doubled to compensate for the degradation of the Y-chromosome to achieve a balance with the autosomes. Although much of the research on dosage compensation is consistent with Ohno’s hypothesis, this hypothesis has been subjected to intense scrutiny and remains controversial. Until recently, many reviews on dosage compensation still take different perspectives^17, 50^.

Most of the studies on dosage compensation have focused on adult somatic tissues. However, by using allele-specific RNA-seq, Marks *et al*.^20^ showed that gene expression of the active X chromosome (Xa) is upregulated, resulting in complete dosage compensation between X-linked and autosomal genes when female ESCs differentiated into EBs. Our results from calculating the dosage compensation in early embryogenesis in mouse were in accordance with this study. We also showed that the average gene expression level from the active X chromosome increased during the inactivation of the other X chromosome.

**Figure 5:**
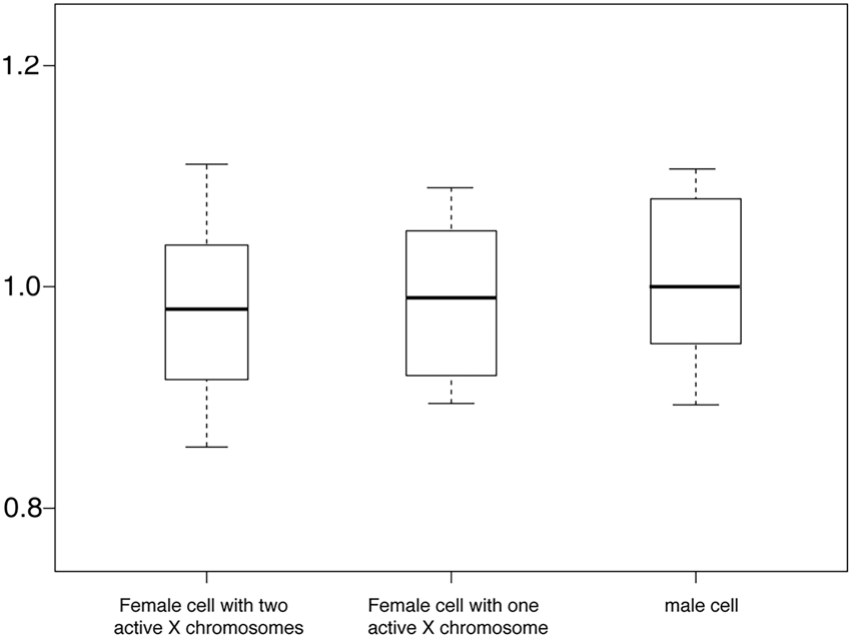
There is a similar X: A expression ratio whether there are one or two active X chromosomes present in cells. We divided all the cells in mouse we examined into three groups (female with two active X chromosomes, female with one active X chromosomes and male), the X: A expression ratio for all the three groups are close to 1 whether there are one or two active X chromosomes present in cells (P > 0.05, by Student’s t-test). The X: A ratio for each group is significant close to 1 compare to 0.5 (student’s t-test, p < 0.001). The percentiles in all boxplots are 0.05, 0.25, 0.5, 0.75 and 0.95.

Marks *et al*.^20^ and Deng *et al*.^12^ showed that female ESCs have a high X: A ratio. However, the high X: A ratio might be independent of the number of active X chromosome in cells, as a high X: A ratio was also observed in cells derived from brain tissue. To confirm this, we reanalyzed the mouse data we examined from Supplementary Table S5. Our results showed that there was no direct relationship between the X: A ratio and number of active X chromosomes (Fig. 5). In addition, the high X: A ratio is significant close to 1 than 2 (student’s t-test, p < 0.001). Therefore, the high X: A ratios in the brain and female embryonic stem cells compared to other tissues is independent of mammalian sex as well as the number of active X chromosome in cells. Additionally, CHIP-seq that measured a higher level of Pol II S5p at the 5′ end of expressed in X-linked genes compared to autosomal genes in brain than in fibroblasts probably reflecting this issue^51^.

Our results provided more reasonable evidence that low-abundance transcripts might result from technical or biological noise rather than active transcripts. Although the effects of low FPKM on the calculation of the X: A ratio has been reported in other papers, there is not enough evidence to confirm that low-abundance transcripts result from technical or biological noise rather than active transcripts. Our results demonstrate that it is inappropriate to calculate the X: A ratio in cells containing two active X chromosomes when all genes are included. It is obviously impossible to observe such a low X: A (XX: AA) ratio in cells containing two X chromosomes. Additionally, we used RT-PCR assay to examine whether these low-expression genes resulted from technical or biological noise and subsequent result showed that 87.5% (35/40) of selected genes were not actively transcripted.

The reason that we calculated the X: A ratios with genes above a value (i.e., 1) was to exclude genes that are not expressed. Previous studies and our results proved that many genes with very small FPKM are actually not expressed and that the small expression values result from improper mapping of reads from homologous sequences. The genes with low levels of expression are much more distributed on the X chromosome (Supplementary Fig. S13), which explains why we obtained a low X: A ratio. We propose that the dosage compensation mechanism to equalize the output between the X chromosome and autosomes is expected to target only genes that are appreciably expressed, as there would be no need for such a mechanism to work on genes that are silent or strongly repressed under the cellular conditions assayed.

Our results from the analysis of RNA-seq data obtained from the process of inactivation/reactivation in both germ cell development and early embryogenesis in mouse suggest that genes expression is balanced between the X chromosomes and autosomes in mouse. More importantly, the SNP-containing RNA-seq data from individual cells of mouse preimplantation embryos of mixed backgrounds provide direct evidence that the gene dosage of the single active X chromosome is upregulated to achieve a similar level to that of two active X chromosomes and autosomes present in two copies. This is perhaps the reason why monosomy for any mammalian chromosome is a hard lethal but the X chromosome as a single active copy is viable.

Wang *et al*.^52^ found a gradually increasing X: A values in mouse male preimplantation embryos, which were not in accordance with our and other finding^6, 53^. We believed that these different conclusions are due to different statistical methods. When calculated X: A ratio, Wang *et al*. calculated the ratio of the average expression level of X-linked genes to that of autosomal genes, however, we and others^6–16, 20^ calculated the ratio of the median expression level of X-linked genes to that of autosomal genes. The advantage that calculated X: A ratio by median could avoid that the expression level is driven by just few highly expressed genes. Another difference of statistical methods being used between Wang *et al*. and us is the single cell nature of the measurements. We test the X: A value for per single cells respectively, then we got the mean X: A value over the cells of the given embryonic day. However, Wang *et al*. calculated X: A ratio with mean FPKM of X chromosome and autosomes in each embryonic day. Our statistical methods actually make use of the single cell nature of the measurements since dosage compensation is a biological phenomenon that happens in single cell level. Furthermore, not only do FPKM cutoffs affect X: A ratio calculation but so do choice of short-read mapping parameters, reference genome annotation, expression data distribution, tissue source for RNA and RNA-seq library construction methods and inclusion of single- vs. multi-copy genes^54^.

The novelty of our observation is that the X: A ratio is same in cell types in which X chromosomes inactivation has not yet taken place and also in cell types with inactive X chromosomes (the X: A ratio in all cell types is about 0.5 if no-/low-expression genes are included in the analysis, and the ratio is close to 1, if no-/low-expression genes are excluded). Our observation implies the existence of a buffering system consisting of some sort of a “sensing mechanism” to know how many X chromosomes are being transcribed, and a mechanism to increase the transcriptional output if there is only one X chromosome being transcribed. The SNP data in the embryo profiles again implies the existence of a buffering mechanism: the maternal X appears more highly expressed in male embryos (where the maternal X is the only X chromosome) than in female embryos (where the paternal X also contributes to expression)–and this mechanism appears present and fully functioning by the 4-cell stage.

Some studies believe that changes in gene dosage during sex chromosome evolution would have greater consequences for some genes (such as dosage-sensitive genes) but weaker consequences for others (such as 1-to-1 orthologs between mammals and birds)^15, 16, 19^. However, our analysis identified the integral dosage balance between X chromosome and autosomes for expressed genes in cells not only with one active X chromosome but also with two active X chromosomes. Therefore, dosage compensation may refer to the integral gene expression balance between X chromosomes and autosomes instead of the idea that gene expression in the active X chromosome is simply doubled one by one. Previous studies showed that inactivation could creep slowly along the mammalian X chromosomes^55–58^, our analysis in the process of inactivation/reactivation indicated that dosage compensation would kept pace with inactivation, the increase in the average gene dosage on the active X chromosome was dependent on the decrease in the gene dosage on the inactive X chromosome.

## Methods

### Animals

We used Mvh-cre (Mvh-cre) male mice^59^ and ROSA^mT/mG^ female mice^60^ (a double-fluorescent *Cre* reporter mouse that expresses membrane-targeted tandem dimer Tomato (mT) before *Cre*-mediated excision and membrane-targeted green fluorescent protein (mG) after excision) to create Mvh-GFP transgenic mice (Mvh-cre;ROSA^mT/mG^). The Mvh*-cre* male mice and ROSA^mT/mG^ female mice were purchased from the Model Animal Research Center of Nanjing University, China. All animal experiments were approved by the Institutional Animal Care and Use Committee of Shanghai and were performed according to the National Research Council Guide for the Care and Use of Laboratory Animals.

### Isolation and purification of FGSCs from mouse ovaries

Ovaries from Mvh-GFP transgenic mice (aged 3–5 days) were collected, washed with ice-cold phosphate-buffered saline (PBS), and cut into small pieces. Two-step enzymatic isolation of FGSCs was performed, as described previously^27^. Briefly, the mice ovarian tissues were treated with 1 mg/ml collagenase (Type IV; Sigma), followed by 0.05% trypsin and 1 mM EDTA digestion at 37 °C to dissociate cells. After passing through a 13-μm nylon cell filter, the cells were suspended in PBS and subjected to FACS, according to the manufacturer’s instructions, to sort GFP-positive cells.

### Isolation and purification of PGCs from mouse genital ridges

The parts of the XX genital ridges (E12.5, E13.5 and E16.5) from Mvh-GFP transgenic mice were dissociated by treating them with 1 mg/ml collagenase (Type IV; Sigma), followed by 0.05% trypsin and 1 mM EDTA digestion at 37 °C to dissociate cells. After passing through a 30-μm nylon cell filter, the cells were suspended in PBS and MVH^+^ cells were separated by FACS, as described above, according to the manufacturer’s instructions.

### Isolation mouse tail fibroblasts

Mouse tails tip were cut, and sprayed with 70% EtOH. After removing the superficial dermis, the tails were cut into 2–3 mm pieces and placed in gelatin coated dishes (35 mm) containing mediun (DMEM with 4.5 g/l glucose and L-glutamine (Gibco), 10% fetal bovine serum, and 1% penicillin/streptomycin). The explants were incubated at 37 °C, 5% CO2, for 5 days, during which fibroblasts migrated out of the explants. The explants were then removed and the cells were cultured in fresh medium until they reached confluence.

### Nongrowing and fully grown oocytes collection

NGO were collected from the ovaries of newborn female mice at 3 days of age. The ovaries were dissociated by treating them with 1 mg/ml collagenase (Type IV; Sigma), followed by 0.05% trypsin and 1 mM EDTA digestion at 37 °C. After pipetting of ovaries, NGO (diameter: 15–30 μm) were collected using a micromanipulator. FGO were collected from the ovaries of 6-week-old C57BL/6 female mouse 46–48 h after 8–10U pregnant mare serum gonadotropin (PMSG) injection. The antral follicles were punctured by 30-gauge needles, and the FGO enclosed by several layers of cumulus cells were selected. Adherent cumulus cells were removed by hyaluronidase treatment, and the denuded oocytes were collected.

### Single-cell RT-PCR

A single cell was incubated in reverse transcription buffer supplemented with 0.1% NP-40 and 0.5 U RQ1 RNase-free DNase (Promega) for 15 min at 37 °C, 3 min at 75 °C, and 5 min on ice. Reverse transcription was carried out by adding 0.5 μl of 100 μM random 6-mer primers, 0.5 μl of 10 mM dNTP mix (Invitrogen), and 0.5 μl of 200 U/μl SuperScript III reverse-transcriptase (Invitrogen). This was followed by incubation at 50 °C for 1 h. The reactions were incubated at 37 °C for 15 min with 1U of RNase H (Invitrogen). The cDNA was then amplified with a Multiple Annealing and Looping Based Amplification Cycles (MALBAC) kit. We carried out two rounds of PCR amplification of the cDNAs to detect *Xist* genes, and two FGSCs and oocyte marker genes (*Mvh* and *Oct4*). For the first-round PCR, we used a mixture of all the primers listed in Supplementary Table S6 to amplify all of the sequences in 20-μl reactions. Aliquots (1 μl) of the first PCR products were used as templates for the second PCR in 20-μl reactions. PCR products were isolated, sub-cloned, and sequenced to confirm the gene sequences.

### RT-RCR

Total RNA exaction from FGSCs and PGCs was performed as previously described^27^. Reverse transcription was performed using a HiScript^®^IIQRTSuperMix (+gDNA wiper) kit (Vazyme, R223-01), according to the manufacturer’s instruction. For RT-PCR, 30 cycles were performed using Taq polymerase (Takara, R10T1M) with primer sets specific for each gene (Supplementary Table S7). Samples were detected using ethidium bromide (EB) staining. PCR products were isolated, sub-cloned, and sequenced to confirm the gene sequences.

### Immunofluorescence

The cells were fixed with 4% paraformaldehyde (PFA) in PBS (pH 7.4) for 15 min at 37 °C. After washing with PBS twice, the cells were permeabilized for 10 min with 0.1% Tween 20 in PBS (PBST). Cells were then incubated in blocking solution (10% goat serum in PBS) for 30 min at 37 °C, before being incubated with primary antibody overnight at 4 °C. Primary antibodies against the following molecules were used: H3K27me3 (1:200; Abcam). After three consecutive 5-min washes in blocking solution, cells were incubated for a further 30 min at 37 °C with secondary TRITC-conjugated antibodies (1:200; Invitrogen) diluted in PBS. The cells were then washed with PBS twice and stained with 4,6-diamidino-2-phenylindole dihydrochloride (DAPI) in PBS. Cells were further washed twice with PBS, then viewed under a fluorescence microscope.

### RNA-seq data and analyses in mouse

To generate expression data for mouse FGSC, E12.5 PGCs and FGO by RNA-seq, purified mRNA was used to build a sequencing library for Illumina sequencing. We sequenced the library by Illumina Hiseq 2500 and aligned RNA-seq reads to Mus musculus UCSC mm10 references with the Tophat software (http://tophat.cbcb.umd.edu/), and calculated the FPKM of each gene using Cufflinks (http://cufflinks.cbcb.umd.edu).

### Gene expression level determination

Gene expression was measured by FPKM (Fragments Per Kilobase Per Million reads). Gene expressions of the mammalian tissues presented in Supplementary Fig. S4 (seven *Rattus norvegicus* tissues, seven *Sus scrofa* tissues, eight *Bos taurus* tissues and eight *Macaca mulatta* tissues) were directly obtained from Baseline Atlas (under Expression Atlas)^61^. The Baseline Atlas provides gene expressions under “normal” conditions. Gene expressions of other tissues, cell lines or single cells were calculated using methods similar to those used by the Baseline Atlas. First, raw sequencing reads were trimmed with Trimmomatic^62^ with the following parameters: ILLUMINACLIP: 2: 30: 10 LEADING: 20 TRAILING: 20 SLIDINGWINDOW: 4: 20 MINLEN: 30. Next, clean reads were mapped to the reference genomes (the hg19 assembly for human and the mm10 assembly for mouse) with Tophat2^63^ with “-p 8 -segment-length 25 -G gene annotation” as the parameters. Finally, the gene expressions were calculated with Cufflinks^64^ with “-p 8 –G gene annotation” as the parameter. Human and mouse Refseq gene annotations were used as the input for Tophat2 and Cufflinks.

### X: A ratio analysis

We calculated the global X: A ratio from the gene expression data. Genes were first grouped into A-genes (autosome) and X-genes (X-chromosome). Only genes with an expression value >Exp (Exp = 0, 1, 2, …) were retained for subsequent analysis. A bootstrapping step was used to estimate the global X: A and its confidence interval. We randomly selected 100 A-genes and 100 X-genes and calculated the median (X-genes)/median (A-genes). This step was repeated 1000 times. The global X: A was estimated as the median of the 1000 values.

### Determining the maternal and paternal expression

Maternal (CAST/EiJ) and paternal (C57BL/6J) expressions were determined in three steps. First, we calculated the gene expression (sum of maternal and parental expression) as described in the “Gene expression level determination” section above. Second, we calculated the ratio of maternal and paternal expression for each gene. We obtained the SNP data from Dr. Rickard Sandberg and for each gene we calculated the number of mapped reads supporting a CAST/EiJ genotype or a C57BL/6L genotype. The ratio of maternal and paternal expression was estimated by the ratio of read numbers supporting a CAST/EiJ genotype or a C57BL/6L genotype. Finally, we assigned the overall gene expression to maternal and paternal expression based on their proportions.

### Accession numbers

Our RNA-seq data (mouse FGSCs, E12.5 PGCs and FGO) in this study has been deposited to gene expression omnibus (GEO) database under accession number GSE75738. Public RNA-seq data were downloaded from the GEO, European Nucleotide Archive (ENA), and DNA Data Bank of Japan (DDBJ) databases. The accession numbers of the datasets are listed in Supplementary Table S8 ^33, 65–71^.

## Electronic supplementary material

supplementary information
