# Supplementary material for: Dosage compensation in the process of inactivation/reactivation during both germ cell development and early embryogenesis in mouse
Source: Sci Rep. 2017 Jun 16;7:3729. doi: 10.1038/s41598-017-03829-z (PMC5473838; doi:10.1038/s41598-017-03829-z)
Supplement: Supplementary file 1 — supplementary information [file 41598_2017_3829_MOESM1_ESM.pdf]

Supplementary information for

Dosage compensation in the process of inactivation/reactivation during both germ cell development and early embryogenesis in mouse

Xiaoyong Li[1¶], Zhiqiang Hu[2,3¶], Xuelin Yu[2,3¶], Chen Zhang[1], Binbin Ma[1]， Lin He[1], Chaochun Wei[2,3*] and Ji Wu[1, 4*]

Supplementary information includes

Supplementary Figure S1- S15.

Supplementary Table S1- S8.

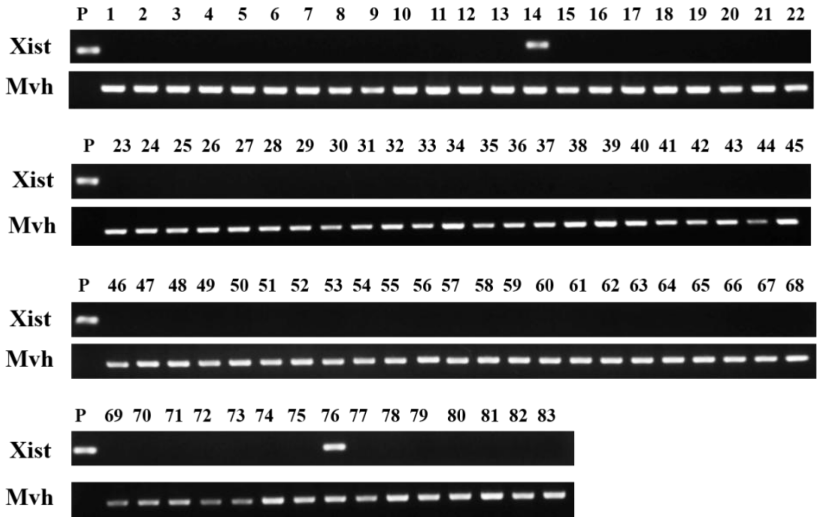

Supplementary Figure S1. Single cell RT-PCR analysis of Xist, Mvh for E12.5 PGCs. Lane P, female tail fibroblasts (positive control); lane 1-83, single cell E12.5 PGCs.

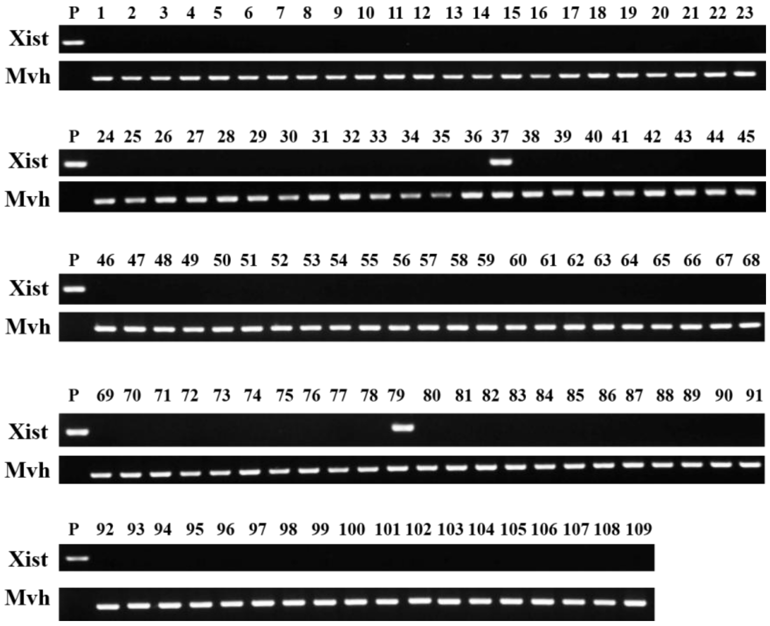

Supplementary Figure S2. Single cell RT-PCR analysis of Xist, Mvh for E13.5 PGCs. Lane P, female tail fibroblasts (positive control); lane 1-109, single cell E13.5 PGCs.

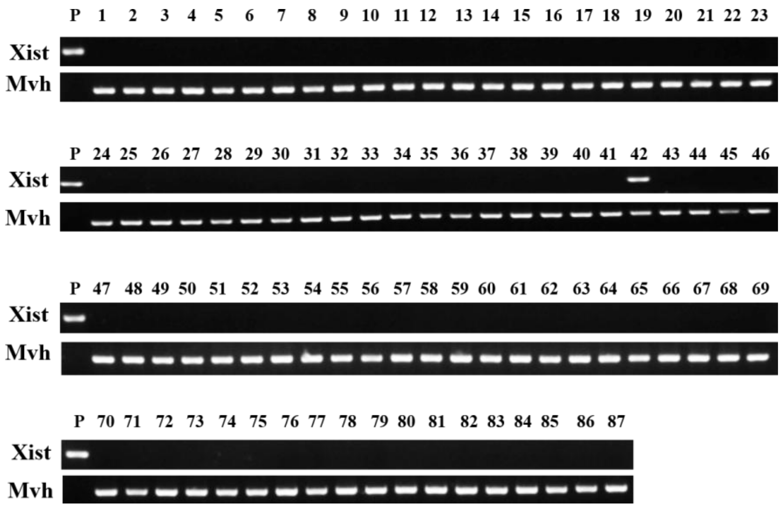

Supplementary Figure S3. Single cell RT-PCR analysis of Xist, Mvh for E16.5 PGCs. Lane P, female tail fibroblasts (positive control); lane 1-87, single cell E16.5 PGCs.

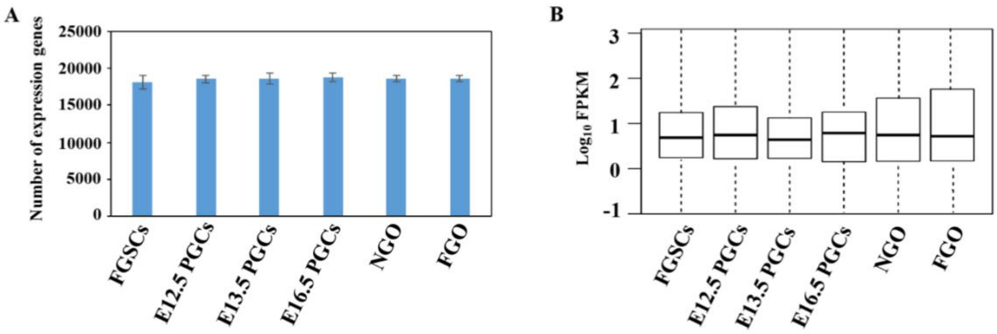

Supplementary Figure S4. There are no statistically significant differences between the RNA-seq data of each types of germ cells. (A) Similar expression gene number between each types of germ cells. (B) Similar gene expression level between each types of germ cells.

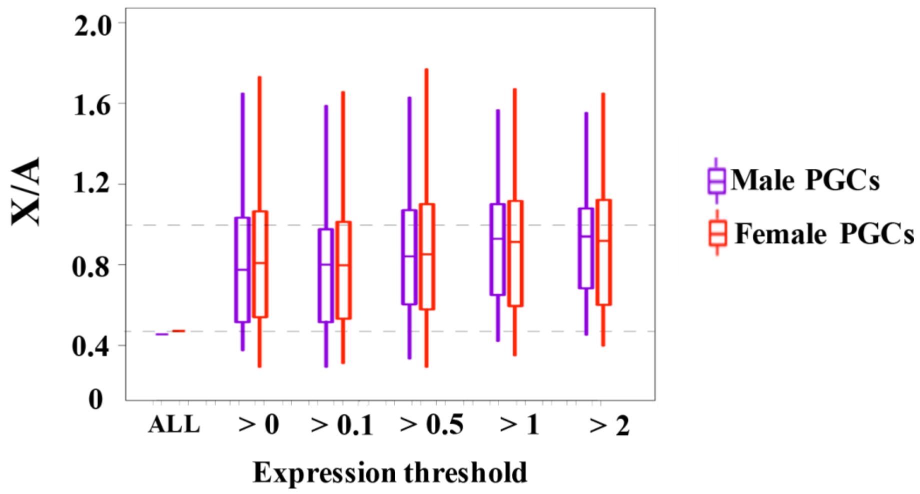

Supplementary Figure S5. Comparisons of X: A ratio between male and female E13.5 PGCs. For both male and female E13.5 PGCs, the X: A median expression ratios increased to close to 1 as no-/low-expression genes were gradually removed and remained stable after genes with FPKM≤1 were removed. There were no diffeences between each types of germ cells. (P > 0.05, by student's t-test).

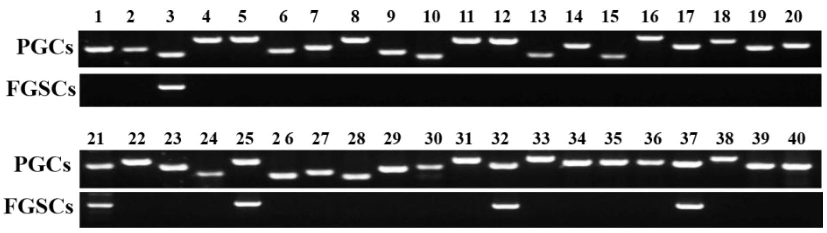

Supplementary Figure S6. Low-abundance transcripts result from technical or biological noise rather than active transcripts. Forty genes with low-expression (FPKM < 1) in FGSCs while high-expression (FPKM > 1) in PGCs were randomly selected from our RNA-seq data. lane 1-40: Pfn4, Cxcl14, Fam3b, Eno2, Fam131c, Rab25, Cd48, Cacnb4, Mmp9, Lama2, Tnfaip6, Xylt1, Omd, Lrriq1, Scd3, Lctl, Nek5, Cldn18, Fank1, Cela1, Gad2, Nppb, Lsamp, Grid1, Exd1, Rab25, Kcnc3, Ica1l, Filip1, Pde4d, Wdfy4, Fzd4, Thsd7b, Ang, Atg9b, Yod1, Slc5a5, Cabp1, Sbp, Spink2.

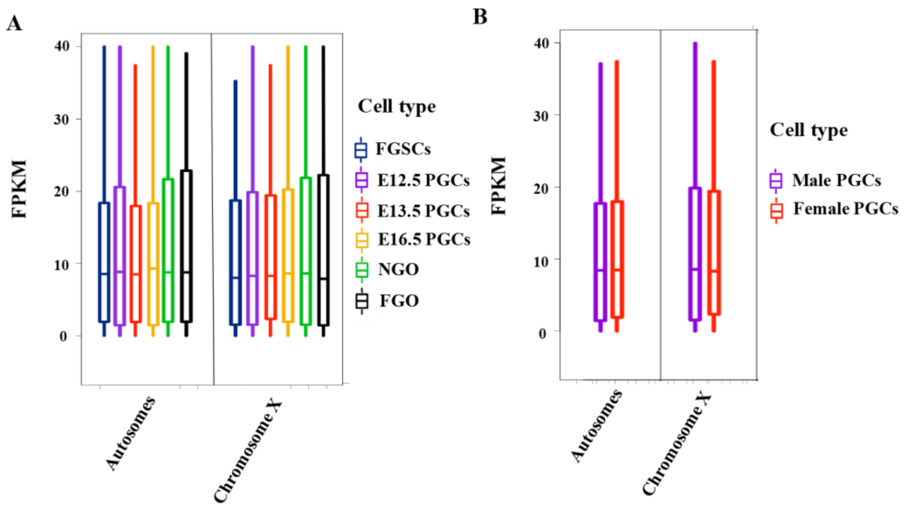

Supplementary Figure S7. Distribution of gene expression across all different samples. A. Distribution of gene expression in germ cell development. B. Distribution of gene expression in male and female E13.5 PGCs. All genes with an expression level of FPKM > 1 are included.

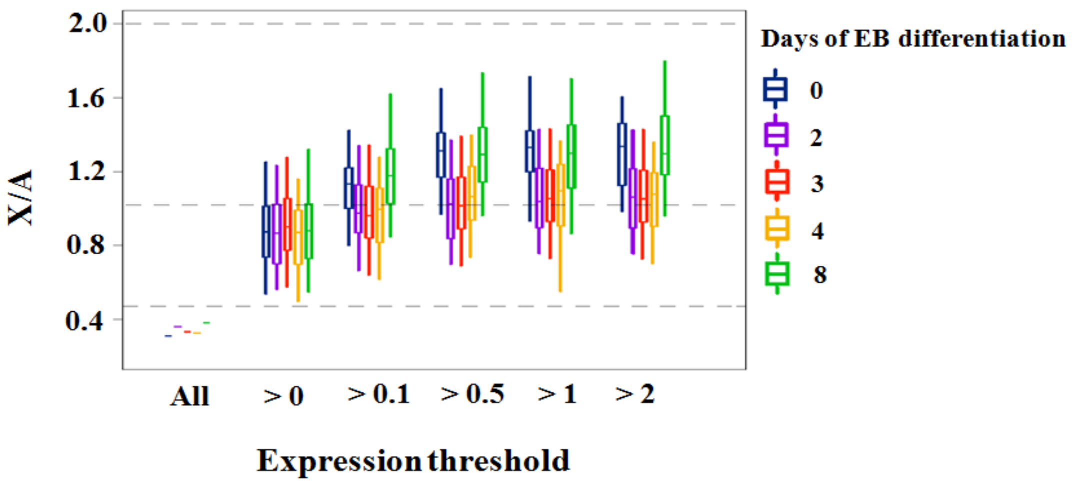

Supplementary Figure S8. X: A median expression ratios during the early stages of XCI by differentiation of female ESCs to EBs were strongly dependent on FPKM cutoffs (>0, ≥0.1, ≥0.5, ≥1, ≥2).

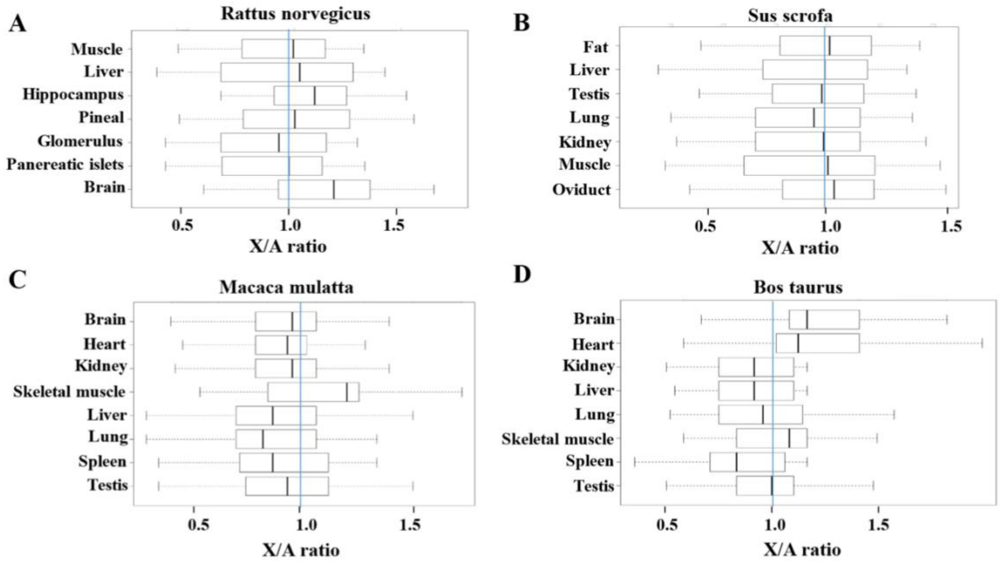

Supplementary Figure S9. Comparisons of RNA-Seq gene expression levels between the X chromosome and autosomes from mammalian tissues (seven *Rattus norvegicus* tissues, seven *Sus scrofa* tissues, eight *Bos taurus* tissues and eight *Macaca mulatta* tissues) by excluding no-/low-expression genes. (A) X: A ratios of median expressions from seven *Rattus norvegicus* tissues. The X: A ratio is significant closer to 1 compared to 0.5 (student's t-test, p<0.001). (B) X: A ratios of median expressions from seven *Sus scrofa* tissues. The X: A ratio is significant closer to 1 compared to 0.5 (student's t-test, p<0.001). (C) X: A ratios of median expressions from eight *Macaca mulatta* tissues. The X: A ratio is significant closer to 1 compared to 0.5 (student's t-test, p<0.001). (D) X: A ratios of median expressions from eight *Bos taurus* tissues. The X: A ratio is significant closer to 1 compared to 0.5 (student's t-test, p<0.001). The percentiles in all boxplots are 0.05, 0.25, 0.5, 0.75 and 0.95.

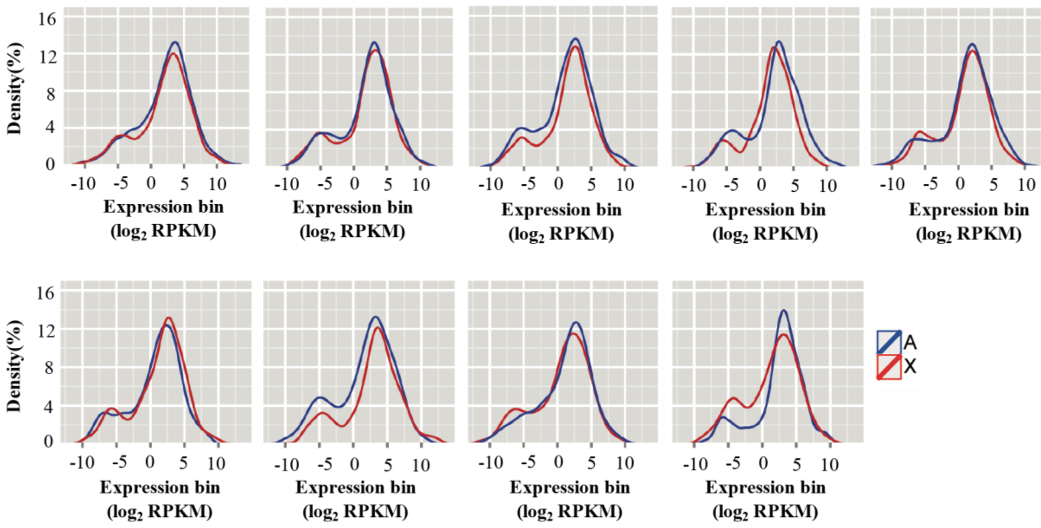

Supplementary Figure S10. Histograms of X-linked and autosomal expression distributions (P >0.05, by Kolmogorov-Smirnov test).

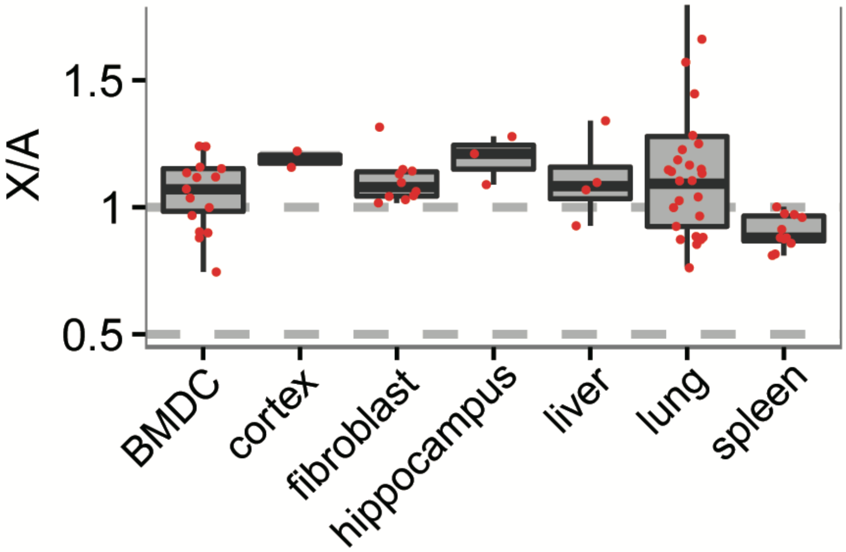

Supplementary Figure S11. X: A ratios of median expressions based on single cell RNA-Seq from bone marrow-derived dendritic cells (BMDC) (male, N=15), cortex cells (N=2), fibroblast cells (female, N=10), hippocampus cells (N=3), liver cells (female, N=4), lung cells(N=25) and spleen cells (male, N=10) when excluding no-/low-expression genes. Sex information is listed in parentheses after the cell names,

and N represents the number of single cell RNA-seq data with this type of cells in the parentheses after the cell names. There were no differences between each types of single cells. (P > 0.05, by student's t-test) except cortex and hippocampus (P < 0.05, by student's t-test). And the X: A ratio is significant closer to 1 compared to 0.5 (student's t-test, p<0.001). The percentiles in all boxplots are 0.05, 0.25, 0.5, 0.75 and 0.95.

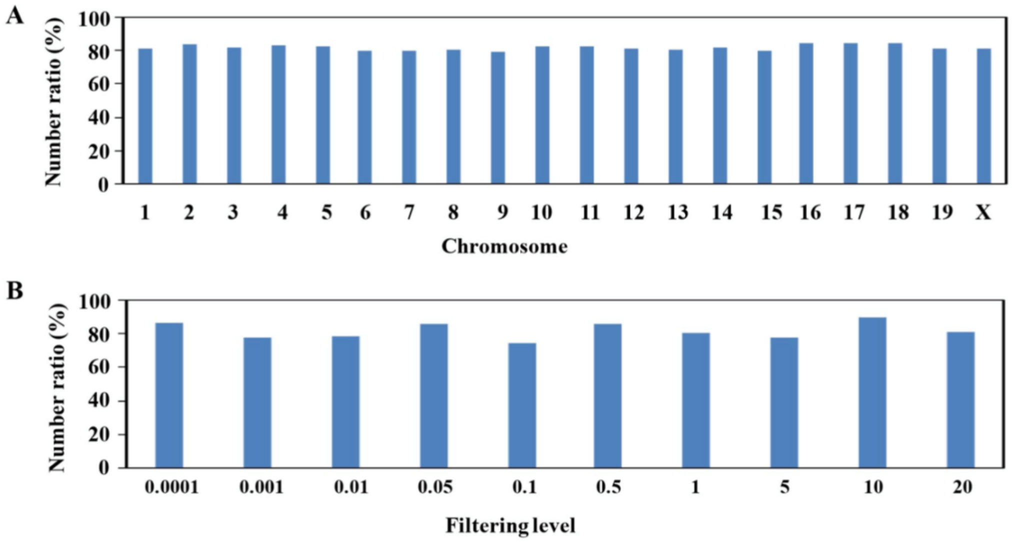

Supplementary Figure S12. The SNPs containing genes were widely scattered on all chromosomes and each level of filtering. (A) Chromosomes are on the X axis, and the distribution ratio is on the Y axis. Vertical bands show the ratio (SNPs containing genes/ total genes) of SNPs containing genes derived from each chromosome. (B) Filtering level are on the X axis, and the distribution ratio is on the Y axis. Vertical bands show the ratio (SNPs containing expression genes/ total expression genes in this filtering level) of SNPs containing genes derived from each level of filtering. In each filter level, our SNP containing genes indicated the remaining number of SNP containing genes after filtering while total genes indicated the remaining number of all ofthe genes (including SNP containing genes and no SNP containinggenes).

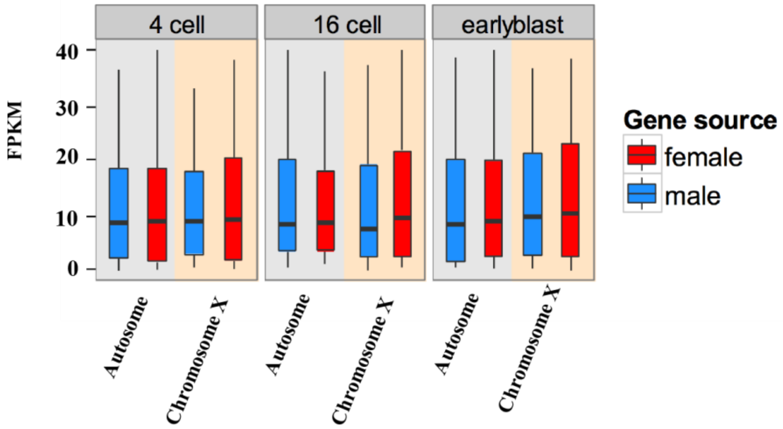

Supplementary Figure S13. Distribution of gene expression across all different samples in mouse early embryogenesis. All genes with an expression level of FPKM > 0.1 are included.

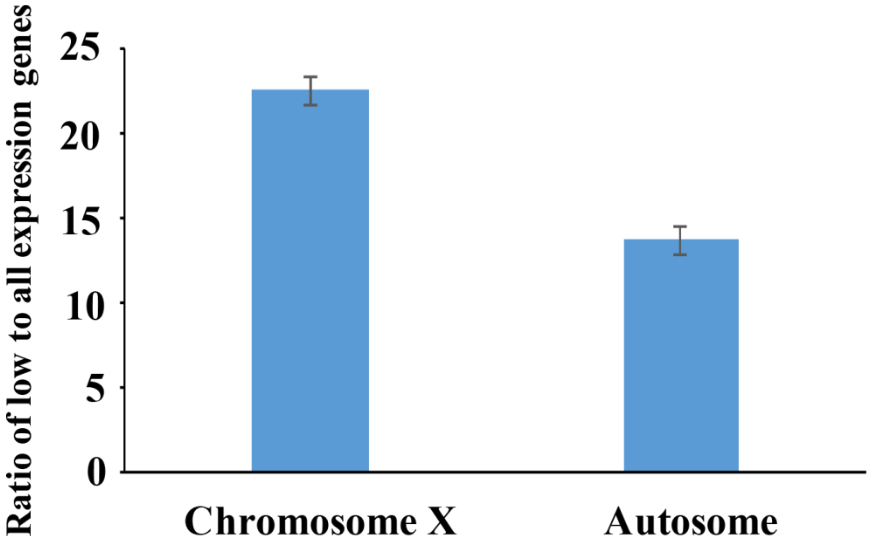

Supplementary Figure S14. The low expressed genes are much more distributed on the X chromosome. We used female E12.5 PGCs as an example.

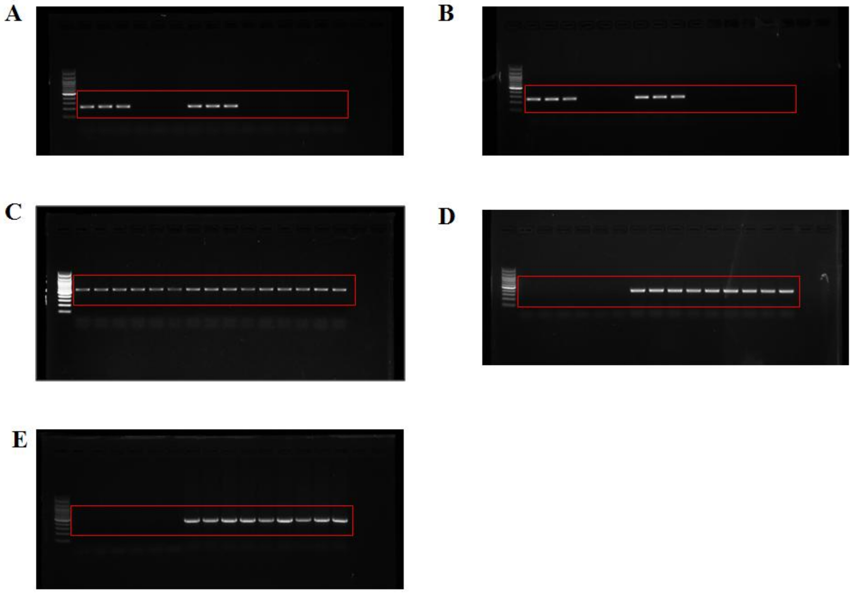

Supplementary Figure S15. Uncropped gels for Figure 1J. (A) Uncropped gels for single cell RT-PCR analysis of Xist ex1. (B) Uncropped gels for single cell RT-PCR analysis of Xist ex2. (C) Uncropped gels for single cell RT-PCR analysis of Gapdh. (D) Uncropped gels for single cell RT-PCR analysis of Mvh. (E) Uncropped gels for single cell RT-PCR analysis of Oct4. Red boxes correspond to those shown in the cropped images within the manuscript.

Supplementary Table S1. The information of RNA-seq data produced in our laboratory

Stages	No. of libraries	No. of cells in each libraries	Total raw reads
FGSCs	2	8	30,333,613
PGCs	2	8	23,107,536
FGO	2	5	29,323,069

Supplementary Table S2. Percentage of expressed genes left after each level of filtering in germ cell development

Cell type	Percentage of genes left after each level of filtering (%)		
	0.1	0.5	1
FGSCs	98.2	92.9	82.5
E12.5 PGCs	98.6	90.1	81.6
E13.5 PGCs	97.3	89.3	80.7
E16.5 PGCs	92.6	88.4	79.8
NGO	96.9	88.1	79.8
FGO	97.2	88.5	80.4

Supplementary Table S3. The median X: A ratio with expression threshold for single cells

Single cells	Access numbers	Expression threshold										
		0.000001	0.0001	0.001	0.005	0.01	0.05	0.1	0.5	1	5	10
BMDC	SRR578548	1.76	1.76	1.76	1.83	1.76	1.73	1.68	1.33	1.3	1.21	1.04
	SRR578549	1.09	1.09	1.09	1.09	1.04	1.19	1.03	1.13	1.12	1.15	1.09
	SRR578550	1.07	1.07	1.07	1.07	1.13	1.17	1.18	1.07	1.18	1.12	1.1
	SRR578551	0.61	0.61	0.61	0.61	0.55	1.07	1.08	1.08	1.24	1.19	1.1
	SRR578552	0.56	0.56	0.55	0.59	0.83	1.28	1.17	1.2	1.2	1.17	1.22
	SRR578553	0.83	0.83	0.83	0.82	0.85	0.77	0.86	1.46	1.3	1.56	1.36
	SRR578554	0.52	0.52	0.52	0.47	0.92	1.72	1.47	1.11	1.26	1.05	0.95
	SRR578555	0.79	0.79	0.77	0.93	0.83	1.07	1.21	1.14	1.28	1	0.94
	SRR578556	1.05	1.05	1.04	0.9	0.76	0.8	1.31	1.11	1.24	1.07	0.91
	SRR578557	1.52	1.52	1.73	1.57	2.05	2.1	1.64	1.43	1.36	1.2	1.16
	SRR578558	0.92	0.92	0.95	0.9	0.9	1.14	1.27	1.14	1.18	1.25	1.15
	SRR578559	0.86	0.86	0.85	1.04	1.16	2.14	1.38	1.39	1.21	1.23	1.23
	SRR578560	1.2	1.2	1.19	1.91	1.72	1.27	0.92	1.01	1.09	1.15	1.26
	SRR578561	2.06	2.06	2.03	2.1	1.95	1.6	1.16	1.13	1.05	1.28	1.23
	SRR578562	1.69	1.69	1.64	1.82	1.76	1.26	1.07	1.12	1.04	1.19	1.24
	SRR578563	0.95	0.95	0.95	0.94	0.93	0.95	0.99	0.79	0.76	1.01	0.97
	SRR578564	0.99	0.99	0.99	0.99	0.98	1	1	1.07	0.95	1.04	0.92
	SRR578565	0.96	0.96	0.96	0.96	0.95	0.96	0.98	1	0.97	1.05	0.92
	SRR578566	0.8	0.8	0.8	0.9	0.93	1.03	1	0.96	1.02	1.11	0.89
	SRR578567	0.96	0.96	0.96	0.96	0.95	1.02	0.99	0.99	1	1.08	0.93
	SRR578568	0.75	0.75	0.75	0.98	0.98	0.99	1.01	0.99	1	1.1	0.91
	SRR578569	0.83	0.83	0.83	0.84	0.83	0.88	0.89	0.95	0.95	1.08	0.9
	SRR578570	0.81	0.81	0.81	0.8	0.8	0.88	0.98	0.99	0.94	1.05	0.93
	SRR578571	0.77	0.77	0.77	0.81	0.8	0.89	0.97	0.96	0.93	1.07	0.92
Cortex	SRR1033341	1.16	1.16	1.17	1.12	1.19	1.28	1.35	1.2	1.24	1.21	1.13
	SRR1033342	1.17	1.17	1.17	1.25	1.34	1.29	1.34	1.35	1.33	1.27	1.6
Fibroblast	SRR1041755	0.62	0.62	0.67	0.67	0.75	0.95	0.88	0.97	1.04	1.15	1.37
	SRR1041756	0.68	0.68	0.7	0.73	0.77	1.06	1.12	1.18	1.15	1.27	1.2
	SRR1041757	0.64	0.64	0.63	0.69	0.72	0.92	0.98	1.1	1.13	1.28	1.13
	SRR1041758	0.76	0.76	0.76	0.76	0.84	0.9	0.95	1.01	1.14	1.08	1.06
	SRR1041759	0.81	0.81	0.82	0.88	0.93	1.11	1.13	1.28	1.32	1.21	1.19
	SRR1041760	1.11	1.11	1.1	1.17	1.19	1.12	1.13	1.07	1.02	0.91	0.92
	SRR1041761	0.75	0.75	0.75	0.85	0.91	1.03	1.07	1.08	1.06	0.99	1

	SRR1041762	0.96	0.96	0.98	1.05	1.06	1.12	1.13	1.13	1.1	1.03	1.05
	SRR1041763	0.98	0.98	0.97	0.96	0.97	0.94	1.05	1.04	1.03	0.93	1.09
	SRR1041764	0.8	0.8	0.8	0.78	0.81	0.88	0.96	1	1.05	1.16	0.99
Hippocampus	SRR1033343	1.2	1.2	1.29	1.29	1.34	1.36	1.33	1.34	1.3	1.31	1.35
	SRR1033344	1.29	1.29	1.29	1.38	1.35	1.37	1.31	1.32	1.32	1.14	1.24
	SRR1033345	1.28	1.28	1.28	1.34	1.31	1.39	1.39	1.29	1.34	1.32	1.27
Liver	SRR805273	0.69	0.69	0.69	0.68	0.8	0.78	0.89	0.97	1.17	1.12	0.91
	SRR805274	0.58	0.58	0.58	0.63	0.85	0.86	1.1	1.09	0.93	0.61	1.21
	SRR805275	0.38	0.38	0.42	0.47	0.6	0.52	0.61	1.63	1.34	1.82	1.26
	SRR805276	0.82	0.82	1.04	1.04	1.04	0.99	0.98	1.27	1.1	0.62	0.74
	SRR805277	0.69	0.69	0.69	0.72	0.75	0.85	0.87	0.97	1.35	1.37	1.34
Lung	SRR1033981	0.97	0.97	0.97	0.97	0.97	0.97	0.97	1.01	1.28	1.07	1.12
	SRR1033982	1.13	1.13	1.13	1.13	1.13	1.18	1.18	1.14	1.44	1.54	1.31
	SRR1033984	0.97	0.97	0.97	0.97	0.97	0.97	0.97	1.07	1.04	1.03	0.96
	SRR1033985	1.21	1.21	1.21	1.21	1.21	1.21	1.21	1.19	1.2	1.04	1.03
	SRR1033986	0.95	0.95	0.95	0.95	0.95	0.95	0.95	0.89	0.85	0.89	0.8
	SRR1033987	0.91	0.91	0.91	0.91	0.91	0.91	0.91	0.91	0.87	1.04	0.98
	SRR1033988	1.23	1.23	1.23	1.23	1.23	1.22	1.39	1.39	1.3	1.3	1.33
	SRR1033989	1.43	1.43	1.43	1.43	1.43	1.43	1.53	1.53	1.53	1.49	1.53
	SRR1033990	1.48	1.48	1.48	1.48	1.48	1.48	1.48	1.43	1.48	1.25	1.26
	SRR1033991	1.32	1.32	1.32	1.32	1.32	1.32	1.32	1.3	1.28	1.18	1.17
	SRR1033992	1.18	1.18	1.18	1.18	1.18	1.18	1.19	1.17	1.15	1.14	1.22
	SRR1033993	1.2	1.2	1.2	1.2	1.2	1.2	1.19	1.42	1.49	1.24	1.27
	SRR1033994	1.23	1.23	1.23	1.23	1.23	1.22	1.24	1.19	1.23	1.22	1.32
	SRR1033995	1.13	1.13	1.13	1.13	1.13	1.13	1.13	1.12	1.1	1.11	1.45
	SRR1033996	0.96	0.96	0.96	0.96	0.96	0.95	1.06	1.19	1.26	1.85	1.56
	SRR1033997	1.07	1.07	1.07	1.07	1.07	1.07	1.07	1.19	1.27	1.16	1.17
	SRR1033998	1.09	1.09	1.09	1.09	1.09	1.08	1.06	1.09	1.31	1.26	1.25
	SRR1033999	0.77	0.77	0.77	0.77	0.77	0.77	0.77	0.78	0.76	0.91	0.82
	SRR1034000	1.25	1.25	1.25	1.25	1.25	1.25	1.25	1.25	1.25	1.17	1.49
	SRR1034001	1.18	1.18	1.18	1.18	1.18	1.18	1.17	1.15	1.13	1.13	1.14
	SRR1034002	1.84	1.84	1.84	1.84	1.84	1.84	1.83	1.81	1.79	1.68	1.52
	SRR1034003	0.97	0.97	0.97	0.97	0.97	0.97	0.97	0.97	0.97	0.95	1
	SRR1034004	1.56	1.56	1.56	1.56	1.56	1.56	1.56	1.64	1.49	2.37	2
	SRR1034006	0.9	0.9	0.9	0.9	0.9	0.9	0.9	0.98	0.92	0.78	0.77
	SRR1034007	1.77	1.77	1.77	1.77	1.77	1.77	1.77	1.74	1.64	1.38	1.58
	SRR1034008	0.96	0.96	0.96	0.96	0.96	1	0.99	0.94	0.87	1.2	1.29
	SRR1034009	1.29	1.29	1.29	1.29	1.29	1.28	1.28	1.16	1.58	1.76	1.78
	SRR1034011	1.51	1.51	1.51	1.51	1.51	1.51	1.51	1.5	1.5	1.34	1.12
	SRR1034012	1.2	1.2	1.2	1.2	1.2	1.2	1.2	1.2	1.15	1.07	1.18
	SRR1034013	1.14	1.14	1.14	1.14	1.14	1.14	1.14	1.11	1.22	1.13	1.16

	SRR1034014	1.14	1.14	1.14	1.14	1.14	1.14	1.14	1.13	1.14	1.16	1.39
	SRR1034015	1.1	1.1	1.1	1.1	1.1	1.1	1.1	1.1	1.07	1.02	1.06
	SRR1034016	1.14	1.14	1.14	1.14	1.14	1.14	1.14	1.14	1.14	1.13	1.08
	SRR1034017	1.27	1.27	1.27	1.27	1.27	1.33	1.31	1.34	1.25	1.25	1.32
	SRR1034018	0.89	0.89	0.89	0.89	0.89	0.89	0.89	0.89	0.88	0.8	0.74
	SRR1034019	1.17	1.17	1.17	1.17	1.17	1.17	1.17	1.14	1.11	1.15	1.27
	SRR1034020	1.47	1.47	1.47	1.47	1.47	1.47	1.45	1.26	1.18	1.31	1.35
	SRR1034021	1.1	1.1	1.1	1.1	1.1	1.17	1.19	1.12	1.17	1.18	1.16
	SRR1034022	1.1	1.1	1.1	1.1	1.1	1.1	1.09	1.01	1.19	1.24	1.16
	SRR1034023	0.82	0.82	0.82	0.82	0.82	0.82	0.82	0.8	0.86	0.92	0.86
	SRR1034024	1.2	1.2	1.2	1.2	1.2	1.19	1.14	1.46	1.3	1.27	1.34
	SRR1034025	0.87	0.87	0.87	0.87	0.87	0.87	0.87	0.87	0.87	0.87	0.83
	SRR1034026	1.02	1.02	1.02	1.02	1.02	1.02	1.02	1.07	1	1.09	0.87
Speen	SRR1106612	0.35	0.35	0.37	0.62	0.71	0.83	0.85	0.93	0.88	0.92	0.92
	SRR1106613	0.47	0.47	0.48	0.64	0.75	0.73	0.72	0.74	0.81	0.96	0.92
	SRR1106614	0.62	0.62	0.65	0.71	0.76	0.8	0.81	0.94	1	0.97	0.95
	SRR1106615	0.61	0.61	0.61	0.68	0.68	0.72	0.73	0.78	0.82	1.23	0.99
	SRR1106616	0.4	0.4	0.4	0.46	0.54	0.71	0.73	0.78	0.76	0.95	1.1
	SRR1106618	0.7	0.7	0.72	0.73	0.73	0.81	0.95	0.93	0.97	1.16	0.98
	SRR1106619	0.73	0.73	0.72	0.76	0.75	0.81	0.79	0.77	0.82	0.93	1.12
	SRR1106620	0.79	0.79	0.78	0.87	0.84	0.85	0.87	0.92	0.88	0.87	0.97
	SRR1106617	0.73	0.73	0.73	0.73	0.72	0.78	0.81	0.87	0.91	1.08	1
	SRR1106622	0.33	0.33	0.37	0.39	0.44	0.58	0.62	0.68	0.78	1.12	1.04
	SRR1106623	0.84	0.84	0.83	0.82	0.81	0.83	0.92	0.96	0.97	0.93	0.95

Supplementary Table S4. Percentage of expressed genes left after each level of filtering in single fibroblast cells

Sample	Percentage of genes left after each level of filtering (%)		
	0.1	0.5	1
SRR1041755	93.1	84.7	78.7
SRR1041756	92.1	82.7	77
SRR1041757	93.9	82.6	78.2
SRR1041758	92.9	82.8	78.9
SRR1041759	93.6	83.2	76.3
SRR1041760	92.2	84.1	79.7
SRR1041761	93.5	84.5	78.6
SRR1041762	93.9	83.1	78.1
SRR1041763	92	82.6	78.7
SRR1041764	92.3	82.6	77.1

Supplementary Table S5. The median X: A ratio of paternal X chromosome (Xp) and maternal X chromosome (Xm) in early embryogenesis

Single cells	Access numbers	Xp/A	Xm/A	X/A	number	Expression level
earlyblas(male)	SRR805294	0	0.89	1.06	14774	0.88
	SRR805295	0	1.07	1.3	14477	0.94
	SRR805296	0	0.89	0.95	14133	0.88
	SRR805297	0	1.14	1.14	14854	0.93
	SRR805298	0	1.07	1.22	14004	0.93
	SRR805299	0	0.85	0.91	15013	0.9
	SRR805300	0	1.07	1.47	15245	0.97
	SRR805301	0	1	1.11	14693	0.94
	SRR805302	0	0.86	0.91	14972	0.94
	SRR805303	0	0.83	0.89	14127	0.95
	SRR805304	0	0.93	0.98	15068	0.89
	SRR805305	0	0.77	0.87	14066	0.91
	SRR805306	0	0.93	1.1	15027	0.92
	SRR805307	0	0.93	1.01	14866	0.9
	SRR805308	0	0.82	1.1	14347	0.88
	SRR805324	0	1.07	1.07	15280	0.91
	SRR805325	0	1.1	1.38	14485	0.88
	SRR805326	0	0.91	1.23	14389	0.89
	SRR805327	0	1.06	1.31	14981	0.96
	SRR805328	0	1.07	1.15	14024	0.96
	SRR805329	0	1.07	1.38	15143	0.91
	SRR805330	0	0.87	0.98	14178	0.89
	SRR805331	0	1.11	1.31	14259	0.89
	SRR805332	0	1	1.34	14826	0.92
	SRR805333	0	1.05	1.26	14601	0.91
	SRR805334	0	1.21	1.33	14987	0.98
	SRR805335	0	0.94	0.99	14065	0.92
	SRR805336	0	1.03	1.16	14064	0.96
earlyblas(female)	SRR805309	0.26	0.83	1.09	15190	0.96
	SRR805310	0.1	0.86	1.1	14952	0.98
	SRR805311	0.02	0.87	1.11	14190	0.9
	SRR805312	0.08	0.8	1.09	14987	0.95
	SRR805313	0.05	0.89	0.97	14679	0.96
	SRR805314	0.3	0.85	1.11	14679	0.88
	SRR805315	0.2	0.75	1.15	15049	0.94
	SRR805316	0.07	0.94	1.26	15358	0.98
	SRR805317	0.2	0.83	1.08	15233	0.95

	SRR805318	0.02	0.89	1.12	14767	0.93
	SRR805319	0.09	0.87	1.18	14957	0.98
	SRR805320	0.14	0.81	1.2	14812	0.89
	SRR805321	0.1	1.02	1.2	14416	0.88
	SRR805322	0.01	1.08	1.29	14015	0.9
	SRR805323	0.19	0.79	1.33	14349	0.95
16cell(male)	SRR805173	0	1.17	1.33	14314	0.9
	SRR805174	0	0.86	0.91	15037	0.95
	SRR805175	0	0.97	1.17	15406	0.89
	SRR805176	0	0.91	0.72	14028	0.95
	SRR805177	0	0.87	0.94	15132	0.89
	SRR805178	0	0.97	1.04	15192	0.91
	SRR805179	0	0.91	0.96	14956	0.96
	SRR805180	0	0.96	0.93	14047	0.88
	SRR805181	0	1.02	1.1	15133	0.98
	SRR805182	0	0.98	1.08	14634	0.97
	SRR805183	0	0.93	0.88	14173	0.93
	SRR805184	0	1	1.18	14947	0.95
	SRR805185	0	0.96	0.85	14557	0.9
	SRR805186	0	0.93	1.1	15434	0.92
	SRR805198	0	1.07	1.22	14496	0.98
	SRR805199	0	0.8	0.81	14768	0.88
	SRR805200	0	0.88	0.98	14424	0.94
	SRR805201	0	0.83	0.86	14835	0.9
	SRR805202	0	0.9	0.98	15226	0.88
	SRR805203	0	0.85	0.95	14785	0.92
	SRR805204	0	0.96	1.06	14505	0.88
	SRR805205	0	0.87	0.97	15261	0.89
	SRR805206	0	0.93	0.53	14288	0.89
	SRR805207	0	0.88	0.85	14949	0.96
	SRR805208	0	0.89	0.84	15027	0.88
	SRR805209	0	1.01	1.05	15006	0.96
	SRR805210	0	0.99	1	15384	0.96
16cell(female)	SRR805189	0.21	0.74	1.18	14886	0.97
	SRR805190	0.1	0.9	1.01	14508	0.98
	SRR805191	0.35	0.54	1.06	14630	0.96
	SRR805192	0.31	0.7	1.15	14430	0.96
	SRR805193	0.28	0.6	1.14	14450	0.98
	SRR805194	0.26	0.63	0.99	15209	0.91
	SRR805195	0.34	0.68	1.19	15043	0.88
	SRR805196	0.27	0.63	1.38	14619	0.89
	SRR805197	0.15	0.81	1.17	14843	0.94
	SRR805211	0.15	0.71	1.01	14509	0.9

	SRR805212	0.16	0.69	1.38	14639	0.92
	SRR805213	0.21	0.64	1.02	14045	0.94
	SRR805214	0.33	0.65	1.04	14015	0.9
	SRR805215	0.19	0.72	1.02	14653	0.96
	SRR805216	0.46	0.69	1.1	14913	0.9
	SRR805217	0.27	0.6	1.04	14223	0.92
	SRR805218	0.27	0.79	1.3	15278	0.92
	SRR805219	0.25	0.68	1.19	14344	0.92
	SRR805220	0.17	0.72	1.24	14991	0.93
	SRR805221	0.31	0.78	1.19	14977	0.95
	SRR805222	0.19	0.8	1.33	14476	0.96
4cell(male)	SRR805223	0	0.85	0.97	15336	0.89
	SRR805224	0	0.95	1.06	14767	0.9
	SRR805225	0.03	0.91	0.94	14624	0.89
4cell(female)	SRR805226	0.49	0.29	1.02	14707	0.91
	SRR805227	0.36	0.49	1.03	14890	0.94
	SRR805228	0.49	0.7	1.01	14887	0.97
	SRR805229	0.47	0.42	1.06	14261	0.96
	SRR805230	0.49	0.47	1.14	14688	0.95
	SRR805231	0.44	0.51	1.18	14137	0.97
	SRR805232	0.63	0.51	0.92	15096	0.94
	SRR805233	0.56	0.35	1.01	14391	0.88
	SRR805234	0.4	0.41	1.02	14890	0.95
	SRR805235	0.48	0.46	0.83	14688	0.88
	SRR805236	0.33	0.49	1.04	15158	0.92

Supplementary Table S6. List of primer pairs used in single cell RT-PCR analyses

Gene	Forward	Reverse	PCR product size (bp)
Xistexon1	CTAAAACTCAGCCCGTTCCA	GCAACCCCAGCAATAGTCAT	217
Xist exon7	GCCCAGGTCACATTATGGTT	CTCCAATTTCTGGGCTCAAG	232
GAPDH	TGGCCTTCCGTGTTCCTAC	GAGTTGCTGTTGAAGTCGCA	568
Oct4	GTTCAGCCAGACCACCATCT	TGGGAAAGGTGTCCCTGTAG	385
Mvh	GCCAGAGGGCTTGATATTGA	CAACTGGATTGGGAGCTTGT	370

Supplementary Table S7.List of primer of 40 genes with low-expression in FGSC while high-expression in PGC

Gene name	Forward	Reverse	PCR product size
Pfn4	CTCACCTTCATAAACCATTCTGTGA	ACAGGGTCTTTTCCTGGAGTTT	311
Cxcl14	ACCAAGAGCATGTCCAGGTA	GGAAGCCTTTCACACACAGC	301
Fam3b	TCAGATCAAGCTGGGTGTTTGT	TCCTTCACAGCTGGACTTCAA	249
Eno2	AGCCCTCATCAGCTCAGGTA	CCCACGTTAGTGGCATCCTT	399
Fam131c	TCTCCTAGCCTGCTCTCCTG	TTGCCCTGGTGTCTGCTTAG	403
Rab25	CCGGACCAGTGCCATCAC	GCCTGTATAGGGTCCAGGTG	262
Cd48	CTGCCCTTGGGAACTGGATT	TTCACGCAGCACTCTCATGT	291
Cacnb4	GTACCTGGAGGCATACTGGC	GCCTATGTCGGGAGTCATGG	380
Mmp9	GAGACTCTACACGGAGCACG	GGAAGACGAAGGGGAAGACG	239
Lama2	AGAGCCATCTGAAGAACACATTGA	ACAACTCTGGGAGGTGGAGTT	200
Tnfaip6	TGCAACCGAAGAGATGGTCG	CCTGTTGAGCCGGATTCCAT	370
Xylt1	TGGGACTTCTTCATCAACCTCA	CCCTGGGGCTTTCCTAGAAC	366
Omd	GTGAAAATGGCTTTACTTCATAAGC	AGTCGTCAGGCTCGGTAGTA	211
Lrriq1	TTGGTGAGTGAGGAGGGTGT	CTCTGAGCTTTGCCTCGCT	300
Scd3	AAGTATGGGCGAGGCTTCCA	CCACTCTTGTGACTCCCGTC	203
Lctl	TGAAGAGCAACATCACCCCC	GGTTGTCGATGTCCACAGGT	390
Nek5	GGTTCTGAAGATTTGTCAAGGACG	CCACCCACGCATGCTTTTTAAT	301
Cldn18	GCTGTACGAGCCCTGATGAT	ATCACTCCCCCAATCAGGGT	350
Fank1	CAACCCGAGCGACGCC	CCCGTGTAAATGACACCGTAG	280

Cela1	GGACGTTCCGGAAACTGACG	CCCGCAAATGTCAACCAGAC	302
Gad2	CCGGGAAGGTCGCCTG	CCTTTATTATTGCTGGTTGCACAG	345
Nppb	GAGGCGAGACAAGGGAGAAC	TACAGCCCAAACGACTGACG	400
Lsamp	AGGGCAGCAATGTAACCCTG	TGTCATCCCGGTACCACTCA	333
Grid1	CATGCACATCCCACACCTCT	CATCCAGACCCAGCCTTGAG	251
Exd1	TTTGGTGTCGCCTTCTCTCC	TGGGTCAGCTACAGAGACGA	380
Rab25	CCCTGGGGAAAAGAGGGC	GCCTGTATAGGGTCCAGGTG	211
Kcnc3	ACAAGAGCCCAATCACTCCC	AACACAGGCTCACTGTTGGG	240
Ica1l	GATGAGGGTGTGACGCCTCG	TTGCTCGTGAGAAGTGACGG	202
Filip1	GGAAGGCGTGAGTCCAGTTAT	GAGCTCACTCGCAACTGGT	279
Pde4d	TGACATGCCTGGAGAATTGGAG	AAGCCACAAAGAGAAAACTGCTT	299
Wdfy4	CCAGATGTGCTAAGCCCACG	ACCAGGGTTAGGAAGCGCAG	380
Fzd4	CCCACAAGACTCCCATCCAG	GCCTACAGTCAGCCGAACAA	320
Thsd7b	CAATGGATGCGCAGAGGTGT	CCGGTTTTCCAGAGGAACTGA	399
Ang	GGGGACGAGATTCCAAAGGA	TTGATGTTGCTCTTGTTGCCA	350
Atg9b	CTGCTGGTTTTGGACGTCAG	AGATGCCCAACCCAAACCTT	350
Yod1	ACCTCTCGCGATGTTTGGAG	CAGCATGTCACCTGACTGGA	349
Slc5a5	CCAGTACCTAGAACTGCGCT	CAGGTTGATCCGGGAATGGT	340
Cabp1	GCTCCCCGCCACAGC	CGGAGCTCTTCAATCTCCTCT	401
Sbp	TCTAAGTGCCAGATTGTACCACA	GCACTGCCCTCTACCTTTATCA	312
Spink2	TGCCGTTACTGGCGGTTCT	AGCACCGGAGGAGAGGAGTT	313

Supplementary Table S8. The accession numbers of the datasets

No.	Cell line or tissue	Database	Access numbers	data source
1	mouse FGSCs	GEO	GSE75738	Our lab
2	mouse E12.5 PGCs	GEO	GSE75738	Our lab
3	mouse E13.5 PGCs	GEO	GSE41908	[64,65]
4	mouse E16.5 PGCs	ENA	ERP001953	[66]
5	mouse NGO	DDBJ	DRP002743	[67]
6	mouse FGO	GEO	GSE75738	Our lab
8	Single BMDC	GEO	GSE41265	[68]
9	Single fibroblast cell	GEO	GSE45719	[33]
10	Single liver cell	GEO	GSE45719	[33]
11	Single lung cell	GEO	GSE52583	[69]
12	Single cortex cell	GEO	GSE52525	[70]
13	Single hippocampus cell	GEO	GSE52525	[70]
14	Single spleen cell	GEO	GSE54006	[71]
15	Single embryonic cell	GEO	GSE45719	[33]
